# Soil seed bank responses to edge effects in temperate European forests

**DOI:** 10.1111/geb.13568

**Published:** 2022-07-16

**Authors:** Cristina Gasperini, Kurt Bollmann, Jörg Brunet, Sara A. O. Cousins, Guillaume Decocq, Karen De Pauw, Martin Diekmann, Sanne Govaert, Bente J. Graae, Per‐Ola Hedwall, Giovanni Iacopetti, Jonathan Lenoir, Sigrid Lindmo, Camille Meeussen, Anna Orczewska, Quentin Ponette, Jan Plue, Pieter Sanczuk, Fabien Spicher, Thomas Vanneste, Pieter Vangansbeke, Florian Zellweger, Federico Selvi, Pieter De Frenne

**Affiliations:** ^1^ Department of Agriculture, Food, Environment and Forestry University of Florence Florence Italy; ^2^ Forest & Nature Lab, Department of Environment, Faculty of Bioscience Engineering Ghent University Melle‐Gontrode Belgium; ^3^ Swiss Federal Institute for Forest Snow and Landscape Research WSL Birmensdorf Switzerland; ^4^ Southern Swedish Forest Research Centre Swedish University of Agricultural Sciences Lomma Sweden; ^5^ Department of Physical Geography Stockholm University Stockholm Sweden; ^6^ UMR CNRS 7058 “Ecologie et Dynamique des Systèmes Anthropisés” (EDYSAN) Université de Picardie Jules Verne Amiens France; ^7^ Vegetation Ecology and Conservation Biology, Institute of Ecology, FB2 University of Bremen Bremen Germany; ^8^ Department of Biology NTNU Trondheim Norway; ^9^ Institute of Biology, Biotechnology and Environmental Protection, Faculty of Natural Sciences University of Silesia Katowice Poland; ^10^ Earth and Life Institute Université Catholique de Louvain Louvain‐la‐Neuve Belgium; ^11^ IVL Swedish Environmental Institute Stockholm Sweden

**Keywords:** deciduous forests, edge effect, forest biodiversity, forest specialists, microclimate, plant traits, soil seed banks, understorey vegetation

## Abstract

**Aim:**

The amount of forest edges is increasing globally due to forest fragmentation and land‐use changes. However, edge effects on the soil seed bank of temperate forests are still poorly understood. Here, we assessed edge effects at contrasting spatial scales across Europe and quantified the extent to which edges can preserve the seeds of forest specialist plants.

**Location:**

Temperate European deciduous forests along a 2,300‐km latitudinal gradient.

**Time period:**

2018–2021.

**Major taxa studied:**

Vascular plants.

**Methods:**

Through a greenhouse germination experiment, we studied how edge effects alter the density, diversity, composition and functionality of forest soil seed banks in 90 plots along different latitudes, elevations and forest management types. We also assessed which environmental conditions drive the seed bank responses at the forest edge versus interior and looked at the relationship between the seed bank and the herb layer species richness.

**Results:**

Overall, 10,108 seedlings of 250 species emerged from the soil seed bank. Seed density and species richness of generalists (species not only associated with forests) were higher at edges compared to interiors, with a negative influence of C : N ratio and litter quality. Conversely, forest specialist species richness did not decline from the interior to the edge. Also, edges were compositionally, but not functionally, different from interiors. The correlation between the seed bank and the herb layer species richness was positive and affected by microclimate.

**Main conclusions:**

Our results underpin how edge effects shape species diversity and composition of soil seed banks in ancient forests, especially increasing the proportion of generalist species and thus potentially favouring a shift in community composition. However, the presence of many forest specialists suggests that soil seed banks still play a key role in understorey species persistence and could support the resilience of our fragmented forests.

## 1 INTRODUCTION

Land‐use changes and forest fragmentation worldwide have rapidly increased the amount of forest edge habitat at a global scale (Riitters et al., 2016; Winkler et al., 2021). As a consequence, 20% of the world's and 40% of Europe's forest area lies within 100 m of a forest edge (Estreguil et al., 2013; Haddad et al., 2015). Therefore, it is crucial to understand how forest edges affect forest biodiversity and functioning.

Forest edges differ greatly from forest interiors in terms of microclimate and are characterized by higher thermal variation, increased solar radiation and higher wind speeds; and thus, increased daily and seasonal temperatures variability, higher light availability and lower soil moisture (Meeussen et al., 2021; Schmidt et al., 2019). Such conditions favour the development of plant communities dominated by species that also occur outside forests, across a wide range of environmental gradients, defined as generalists (Marinšek et al., 2015). By contrast, the shady, humid and more stable microclimate of forest interiors prevents or limits their success (Gasperini et al., 2021; Normann et al., 2016) and is usually more suitable for shade‐tolerant species, referred to as forest specialists (Marinšek et al., 2015). Forest edges are also characterized by a higher seed influx of non‐forest species (Devlaeminck et al., 2005), and higher nitrogen deposition and carbon stocks (Remy et al., 2016), compared to forest interiors. Evidence from previous studies has shown that the magnitude and depth of the edge effect vary with macroclimate (i.e. with larger offsets between forest and free‐air temperature in warmer climates) and with forest structure [with stronger negative effects in open forests: e.g. for the species richness the distance of the edge influence (DEI) is 35.5 m in open forests and ≈ 0 in dense forests] (Govaert et al., 2020; Meeussen et al., 2021). Therefore, it is important to adopt a multiple factor approach to assess how steep micro‐environmental gradients in forest edges may impact plant community diversity and composition.

Defining a vegetation community based uniquely on its present aboveground species composition is incomplete, as there may be many other species waiting to emerge following disturbance (Plue, De Frenne, et al., 2017). Belowground communities of dormant, viable seeds, that is, the so‐called soil seed bank, permit species coexistence in plant communities through the storage effect, extending the size of the plant community (Chesson, 2000). Decades of work demonstrate that these stored communities account for a considerable part of the plant diversity in forests, often differing from the vegetation, both in terms of the presence and relative abundances of species (Larson & Suding, 2022; Plue, De Frenne, et al., 2017). The forest soil seed bank is composed of many herbaceous early successional species (Bossuyt & Hermy, 2001) as well as typical forest herbs (Plue et al., 2013), while trees and shrubs are in general poorly represented (Warr et al., 1994). The composition of soil seed banks depends largely on the historical trajectories and on the abundance of the aboveground vegetation, on climate and on physical and chemical soil properties as well as a variety of other interacting environmental factors (Heydari et al., 2013; Kůrová, 2016). Moreover, soil seed banks, as biodiversity reservoirs (Plue et al., 2021), can help maintain plant species and genetic diversity by buffering species extinctions and genetic erosion (Faist et al., 2013). Given their importance to ecosystem functioning, it is crucial to understand how soil seed banks respond to environmental variation (Faist et al., 2013).

Edge effects may impact macro‐ and micro‐scale patterns in soil seed bank composition and diversity (Lin et al., 2006), generally leading to a progressive reduction of forest species (Lin et al., 2006; Rooney et al., 2004) and accelerating the homogenization of understorey plant communities (Büchi & Vuilleumier, 2014; Staude et al., 2020). The amount of habitat and fragmentation can have a considerable effect on understorey diversity (Watling et al., 2020). For instance, the amount of forest habitat present in the ‘local landscape’ is known to affect specialist species abundance (Watling et al., 2020) due to the low colonization capacity of many forest specialists (Whigham, 2004). Directional changes in community composition can be summarized also via species' thermal niches into a floristic temperature index, which is a community weighted mean of species' temperature preferences (Vangansbeke et al., 2021). Changes in the relative abundance of warm adapted versus cool adapted species can be used as an indicator of the impacts of local temperature change on communities (Zellweger et al., 2020), and can also be applied to reveal cooler temperature preferences of seed bank communities in the interior than in the edge (Gasperini et al., 2021).

Biodiversity changes and functioning are not fully captured by species diversity metrics (Santini et al., 2017). For this purpose, adopting a trait‐based approach can add relevant information and help to address how the edge effect influences the soil seed bank communities. In contrast to realized vegetative communities, the trait‐based tools and approaches familiar to functional ecology are rarely applied to processes shaping seed bank composition and structure – an oversight that could compromise our understanding of the understorey vegetation (Larson & Suding, 2022). Here, traits related to resource acquisition, such as plant height and specific leaf area (SLA), and regeneration, such as seed mass, are known to drive major ecosystem functions and population responses (Bernhardt‐Römermann et al., 2008), and to respond to changing environmental conditions, that is, temperature and light (Maes et al., 2020). The functional profile of European forest understoreys, for instance, varies with latitude but not with the distance to the forest edge, despite significant edge versus interior differences in species composition and diversity (De Pauw et al., 2021). However, whether this finding reflects the lack of a microclimate effect on the aboveground vegetation trait profile, and whether this also applies to the soil seed bank community remains unknown. Because of the significant seed bank species composition changes from the edge to the forest interior (Gasperini et al., 2021), a parallel change of its functional profile can also be expected.

In the forest understorey, variations in the environment driven by latitude, elevation, forest management, edaphic conditions and distance to the forest edge have been shown to influence forest plant communities (Govaert et al., 2020; Pellissier et al., 2013). So far, however, no large‐scale germination trials have focused on the variation of forest soil seed banks between the forest edge versus interior across these environmental gradients, or on common trends in aboveground vegetation and soil seed bank composition. Moreover, little is known about how climate and soil conditions can affect the density, richness, composition and functional traits of soil seed banks across ancient temperate forests at the continental scale. Ancient forests are those that were continuously forested during at least the last two centuries or since the first available land‐use maps and thus not converted to any other land‐use type (Bossuyt & Hermy, 2001). Thus, we aimed to disentangle the edge effect on soil seed bank density, diversity, composition and functional traits along latitudinal, elevational and forest management type gradients in multiple European regions, also accounting for local edaphic, climatic and landscape conditions. Accordingly, we expected that:

1. Seed bank density and diversity are higher at forest edges, with a decreasing proportion of generalist species towards the interior;
2. Local edaphic, macro‐ and microclimatic and landscape conditions influence the soil seed bank density, diversity, composition and functional traits;
3. Species richness in the soil seed bank and the aboveground vegetation are positively correlated and their relationship is affected by microclimatic and forest structural differences between edge and interior;
4. The soil seed bank trait profile differs between edge and interior as a result of variations in species diversity and composition.

## 2 METHODS

### 2.1 Experimental design

Soil sample collection and vegetation surveys were performed in 90 plots (6 regions with only lowland forests plus 3 regions with 3 elevation classes [6 + (3 × 3)] = 15 × 3 forest densities × 2 plots per site) distributed in temperate deciduous forests along a latitudinal gradient of 2,300 km. The gradient stretched from central Italy to central Norway and covered nine regions (Italy, Switzerland, France, Belgium, Poland, Germany, southern Sweden, central Sweden and Norway), thus crossing the sub‐Mediterranean, temperate and boreo‐nemoral climate zones (Supporting Information Figure S1). In three regions, Norway, Belgium and Italy, soil samples were additionally collected along an elevational gradient (low, intermediate and high elevation) while the sampling was restricted to lowland forests for the six other regions. In all regions, and at each elevation, soil samples were also collected in three forest density classes: dense, intermediate and open forests, resulting from different management types. Dense forests (not thinned for at least 10–30 years) are complex forests with high basal area (30 ± 14.7 m^2^/ha), high cumulated sums of canopy cover across the shrub and tree canopy layers (> 120%) and a well‐developed shrub layer. Intermediate forests (regularly thinned with the most recent thinning 5–10 years ago), have a similar basal area (30 ± 16.4 m^2^/ha) but lower canopy cover (100–120%) and a sparse or absent shrub layer. Open forests (regularly thinned and most recently ~4 years before sampling) have a lower basal area (20 ± 10.9 m^2^/ha), lower canopy cover (< 100%) and no shrub or subdominant tree layer. The selected forests are mesic deciduous forests dominated by oak species (mainly *Quercus robur*, *Quercus petraea*, *Quercus cerris*) as well as other deciduous tree species such as *Fagus sylvatica*, *Betula pubescens*, *Populus tremula*, *Ulmus glabra*, *Alnus incana* and *Carpinus betulus*. Within each site (*n* = 45), soil samples were collected in two 3 m × 3 m plots: one at the outermost line of tree trunks (forest edge), and one at 97–100 m perpendicularly from the forest edge (forest interior). A detailed site description is given in Supporting Information Table S1. All forest edges were south‐facing, bordered by grassland or arable land and were created by ancient deforestation (< 1950). For more information about plot selection and experimental design see Govaert et al. (2020).

### 2.2 Herb layer survey

In each 3 m × 3 m plot, all vascular plant species were identified and their percentage of ground cover was estimated (as continuous variables) between May and July 2018. Vegetation surveys were conducted at the peak of vegetation biomass, and the timing followed a latitudinal pattern (southern regions were sampled first, mid‐May; northern regions last, early July). The floristic surveys of the herbaceous layer included all vascular plant species, both woody and non‐woody plants smaller than 1 m, as well as lianas (Supporting Information Table S2). For more information about herb layer surveys see Govaert et al. (2020). Species nomenclature follows Euro+Med (2006).

### 2.3 Soil seed bank sampling

To analyse the persistent soil seed bank (seeds viable > 1 year; Thompson et al., 1997), soil samples were collected between April and May 2020, before the seed rain of most species. Soil samples were collected after at least 3 days of dry weather, within each 3 m × 3 m plot, using soil corers of 5 cm deep × 3.5 cm in diameter (litter removed). Soil cores were taken systematically at *c*. 40 cm from each other, for a total of 49 soil cores per plot, which were pooled together to have one single soil seed bank sample per plot (*c*. 1.2 L of soil in total). Seed bank samples were stored in bags in a dry, cool and dark place until posting to Florence; where they were immediately placed in a greenhouse (43°.47' N, 11°.20' E, 320 m a.s.l.), to start the germination trial. Soil samples were sieved with a 4‐cm sieve and spread out on top of 10 cm sterilized potting soil in plastic pots of 50 cm × 50 cm, with the sampled soil layer always *c*. 0.5 cm deep. Ten control pots filled only with sterilized potting soil were set up alongside the experimental pots to check for contamination (no contamination occurred). The pots were labelled and positioned in the greenhouse with a natural sunlight regime, with day‐ and night‐time temperatures between 12–38 and 2–20 °C, respectively. The irrigation was done through a fog system, that kept the soil moist throughout the trial. The emerging seedlings of all vascular plant species were identified, counted and removed. Seedlings that were difficult to identify were transplanted in larger pots until identification was possible. The experiment ended after 40 weeks when no new seedlings emerged for two consecutive weeks.

### 2.4 Environmental variables

#### 2.4.1 Edaphic properties

In every plot, we randomly collected five topsoil samples after removal of the litter layer (0–10 cm depth, diameter 30 mm) and five subsoil samples (10–20 cm depth) that were pooled for chemical analyses (pH and C : N) and for texture analyses (% sand), respectively. The samples were analysed for pH‐H_2_O by shaking a 1:5 ratio soil/H_2_O mixture for 5 min at 300 revolutions per minute and measuring with an Orion 920A pH meter with a Ross sure‐flow 8172 BNWP pH electrode model (Thermo Scientific Orion, USA). To measure the total concentration of C and N in the soil, the soil samples were dried to constant weight at 40 °C for 48 hr, sieved over a 2‐mm mesh and combusted at 1,200 °C. The gases were measured by a thermal conductivity detector in a CNS elemental analyser (vario Macro Cube, Elementar, Germany). To assess the soil texture (% sand), the soil samples were oven‐dried to constant weight at 40 °C for 48 hr, sieved and sedimented with a Robinson‐Köhn pipette according to ISO 11277 (2009). The litter quality was determined based on tree and shrub species scores (1 = very low quality, 5 = very high quality) and was calculated at plot level. The litter quality describes the quality of the litter and thus the rate of leaf litter decomposition. Very low litter quality (scores close to one) indicates slow decomposition rates. High litter quality (scores close to five) denotes a high decomposition rate. The litter quality scores for each tree and shrub species were derived from Verheyen et al. (2012), Maes et al. (2019) and Vanneste et al. (2020). Scores for species that were not included in any of these sources (e.g. *Juniperus communis*, *Phillyrea latifolia*, *Pyrus communis*) were derived from literature on the litter quality or decomposition rates of those species. Scores for individual tree and shrub species can be found in Supporting Information Table S3.

#### 2.4.2 Forest structure and landscape conditions

Differences in forest structure were quantified using the plant area index (PAI) based on terrestrial laser scanning (TLS). PAI was calculated as the integral of vertically resolved plant area per volume density (m^2^/m^3^) profiles derived from a single‐scan position using a RIEGL VZ‐400 (RIEGL Laser Measurement Systems GmbH, Horn, Austria) in the centre of each plot. PAI indicates the density and complexity of the forest structure and is negatively related to light availability on the forest floor. To estimate the amount of forest habitat in the landscape, the forest cover was calculated as a percentage area with a tree cover > 20% within a radius of 500 m based on satellite‐based global tree cover data with a spatial resolution of 30 m (Hansen et al., 2013).

#### 2.4.3 Macro‐ and microclimate

The mean annual microclimatic soil temperature was derived from lascar temperature loggers (EasyLog EL‐USB‐1, accuracy at −35 to +80 °C: ± 0.5 °C) placed at the centre of each plot. The loggers were buried in the ground in a protective plastic tube at a depth of 5 cm. Soil temperatures were recorded at hourly intervals (2018–2020). The mean annual precipitation was derived from the CHELSA database for 1979–2013 (30‐arcsec resolution; ~1 km^2^; Karger et al., 2017). Mean and standard deviations of the eight predictor variables (% of sand + soil pH + C : N + litter quality + plant area index + microclimate soil temperature + annual precipitation + % forest cover) are given for the nine study regions in Supporting Information Table S4.

### 2.5 Data preparation

First, seed density was calculated as the total number of seedlings that emerged in each pot. Seed density was calculated without *Juncus effusus* because of the very high frequency of this species, which would have confounded the results. Next, species diversity was quantified as species richness and Shannon diversity of the seedling community in each pot.

The species were assigned to five forest guilds following Heinken et al. (2019): species mainly found in closed forests (1.1); species typically found in openings and forest edges (1.2); species occurring in both forest and open habitats (2.1); species occurring less frequently in forest, mainly in open habitats (2.2) and ‘open habitat’ species (O) (Supporting Information Table S5). Mediterranean species that were not included in Heinken et al. (2019) were classified based on Pignatti et al. (2017–2019) and on expert evaluation. The species were grouped into two main categories, forest specialists (1.1 + 1.2 groups) versus generalists (2.1 + 2.2 + O groups). Only for 22 species (7% of the total species) there were no indications about habitat preferences and thus, these species were excluded from the analyses (92 individuals in total, including individuals only determined at genus level and unidentified individuals). Data were not available for Switzerland and thus the species were assigned to each category following the closest region (France).

The community temperature index per plot was calculated as the mean of the temperature preferences based on the ClimPlant database (Vangansbeke et al., 2021) of each species recorded as seedlings in the soil seed bank weighted by their abundance (number of emerged seedlings); for the herbaceous layer, the mean of temperature preferences was weighted by species cover. Woody species were excluded from these analyses (Supporting Information Table S6) because their temperature values may vary greatly during their life cycle (Caron et al., 2021). Thermal niche data were not available for 69 species (out of 257 species; 239 excluded observations out of 1,015), which were excluded from the analyses.

Three functional traits were selected according to the leaf–height–seed (LHS) plant ecology strategy scheme (Westoby, 1998): specific leaf area (SLA: light‐capturing area deployed per dry mass allocated), plant height (height of the plant's canopy at maturity) and seed mass. Trait values were calculated as the mean of the specific trait of each soil seed bank species weighted by its abundance (number of seedlings; hereinafter referred to as CWM) and for each species in the herbaceous layer, the mean of the specific trait was weighted by its plant cover. Species‐specific trait values were derived from different databases: the LEDA trait database (Kleyer et al., 2008); BiolFlor (Kleyer et al., 2008); Kew Seed Information Database (Royal Botanic Gardens Kew, 2021); Brot2.0 (Tavşanoğlu & Pausas, 2018) and TRY (Kattge et al., 2020). Trait values for seed mass, plant height and SLA data were not available for eight species, which were thus excluded from the subsequent analyses (Supporting Information Table S8). However, trait values were available for all the species that occurred in more than 5% of the plots. In total, 70 individuals (belonging to 11 genera) were not identified to species level and thus were excluded from these analyses (community temperature index and CWM of plant traits), as well as 22 unidentified individuals.

The herbaceous layer was analysed for each plot following the same methodology described for the soil seed bank (unless otherwise specified).

### 2.6 Data analysis

All analyses were performed in the R environment (R Core Team, 2021). Linear mixed‐effects models (LMMs) and generalized linear mixed‐effects models (GLMMs) were fitted using the packages ‘lme4’ (Bates et al., 2015) and ‘MuMln’ (Bartoń, 2019). Soil seed bank density, species richness, Shannon diversity, generalist and specialist richness, the proportion of specialists, CWM seed mass, height and SLA, and the community temperature index were tested as response variables. Mean and standard deviations of the response variables are given for the nine study regions in Supporting Information Table S9.

All the response variables were calculated for each of the 90 plots. To ensure normality, the seed mass was log‐transformed. In all models, region and transect nested within region were added as random intercept terms in mixed‐effect models (as 1 |region/transect in R syntax) to account for spatial autocorrelation that may arise from the hierarchical structure of the data. All continuous predictor variables were scaled (*z*‐transformation) to allow the comparison of model coefficients. In a first set of models (1), the fixed effects were the four gradients of the experimental design [latitude, elevation, forest type and (edge versus interior)]. In a second set of models (2) the fixed effects were eight continuous environmental variables representing variation in climate, soil and litter properties, and forest structure: % of sand, pH of the mineral soil, C : N ratio, litter quality, PAI, % canopy cover, soil temperature and mean annual precipitation. Finally, a third set of models was assessed to test the influence of the four design variables [latitude, elevation, forest type and (edge versus interior)] on the eight environmental variables considered in the second set of models (Supporting Information Table S11). For the sake of simplicity, no interactions were considered.

As for the different studied response variables: a negative binomial distribution was used for seed density as this kind of count data was overdispersed; a Poisson error distribution with a log link function was used for the other count data (species richness, generalist richness, specialist richness); while a binomial distribution was adopted for the proportion of forest specialists on total species richness. For all other response variables, a Gaussian distribution was applied, resulting in linear mixed‐effect models (LMMs). The following equations represent the starting models:

(1) $$\begin{array}{c} \text{Response variable}∼\text{latitude}+\text{elevation}+\text{forest type}\\ +\text{edge versus interior}+(1\;|\;\;\text{region}\;/\;\text{transect}) \end{array}$$

(2) $$\begin{array}{c} \text{Response variable}∼\%\text{sand}+\mathrm{pH}+C:N\;\text{ratio}+\text{litter quality}\\ +\text{soil temperature}+\text{annual precipitation}\\ +\mathrm{PAI}+\%\text{forest cover}+(1\;|\;\text{region}\;/\;\text{transect}) \end{array}$$

Starting from these models with all predictor variables the single best model was selected based on the lowest Akaike information criterion (AIC) and Bayesian information criterion (BIC). A selection of models with delta AIC <2 was displayed with the *dredge*‐function of package MuMln (Bartoń, 2019; Fieberg & Johnson, 2015). In all models, residuals were evaluated for normality and homogeneity by a visual check of the model assumptions (normality of residuals, normality of random effects, homogeneity of variance). Multicollinearity of the predictor variables in the models was assessed using variance inflation factors (VIFs). For all models, VIFs among the set of predictor variables we used were smaller than 3.5 and thus no strong multicollinearity issues were detected (Zuur et al., 2009). For all models, we computed the proportion of variance explained by the fixed effects of the model (marginal *R* ^2^) and by both random and fixed effects (conditional *R* ^2^) (Nakagawa & Schielzeth, 2013).

Due to non‐normal distribution of the data, the correlation between the herb layer and the soil seed bank species richness was tested with the Spearman rank correlation coefficient, using the cor.test function of package ‘stats’.

Differences in community composition among regions were quantified with the *vegdist*‐function of the R package ‘vegan v2.4–7’ (Oksanen et al., 2020) using the Raup–Crick distance and visualized with non‐metric multidimensional scaling (NMDS) (*metaMDS*‐function in 'vegan'). Differences were tested using permutational multivariate analysis of variance (PERMANOVA; *adonis*‐function in vegan; 999 permutations with strata = region). Species vectors were fitted using the *envfit*‐function ('vegan'). Multivariate homogeneity of dispersion was also tested using *betadisper* (vegan), restricting permutations by region. Also, Raup–Crick distances were used to quantify the average community composition dissimilarities between edge versus interior within each region. Differences between region in the edge versus interior distance were tested with one‐way ANOVA with the *aov*‐function followed by a post‐hoc Tukey's honestly significant difference (HSD) test with the ‘*TukeyHSD*’ function (‘stat’ package).

## 3 RESULTS

In total, 10,108 seedlings of 250 species covering 138 genera emerged from the soil seed bank (Supporting Information Table S2) plus 11 taxa that could only be identified to the genus level (*Agrostis*, *Carex*, *Cistus*, *Cytisus*, *Convolvulus*, *Eragrostis*, *Juncus*, *Medicago*, *Rubus*, *Salix*, *Trifolium*). The corresponding herb layer included a total of 295 vascular plant species (Supporting Information Table S2) plus 13 taxa only identified to the genus level. The most abundant species in the soil seed bank were *Juncus effusus* (4,458 seedlings; found in 17 out of 90 plots), *Urtica dioica* (510 in 22 plots), *Hypericum perforatum* (334 in 31 plots), *Betula pendula* (331 in 26 plots) and *Deschampsia cespitosa* (269 in 11 plots).

### 3.1 Soil seed bank density and diversity

**FIGURE 1:**
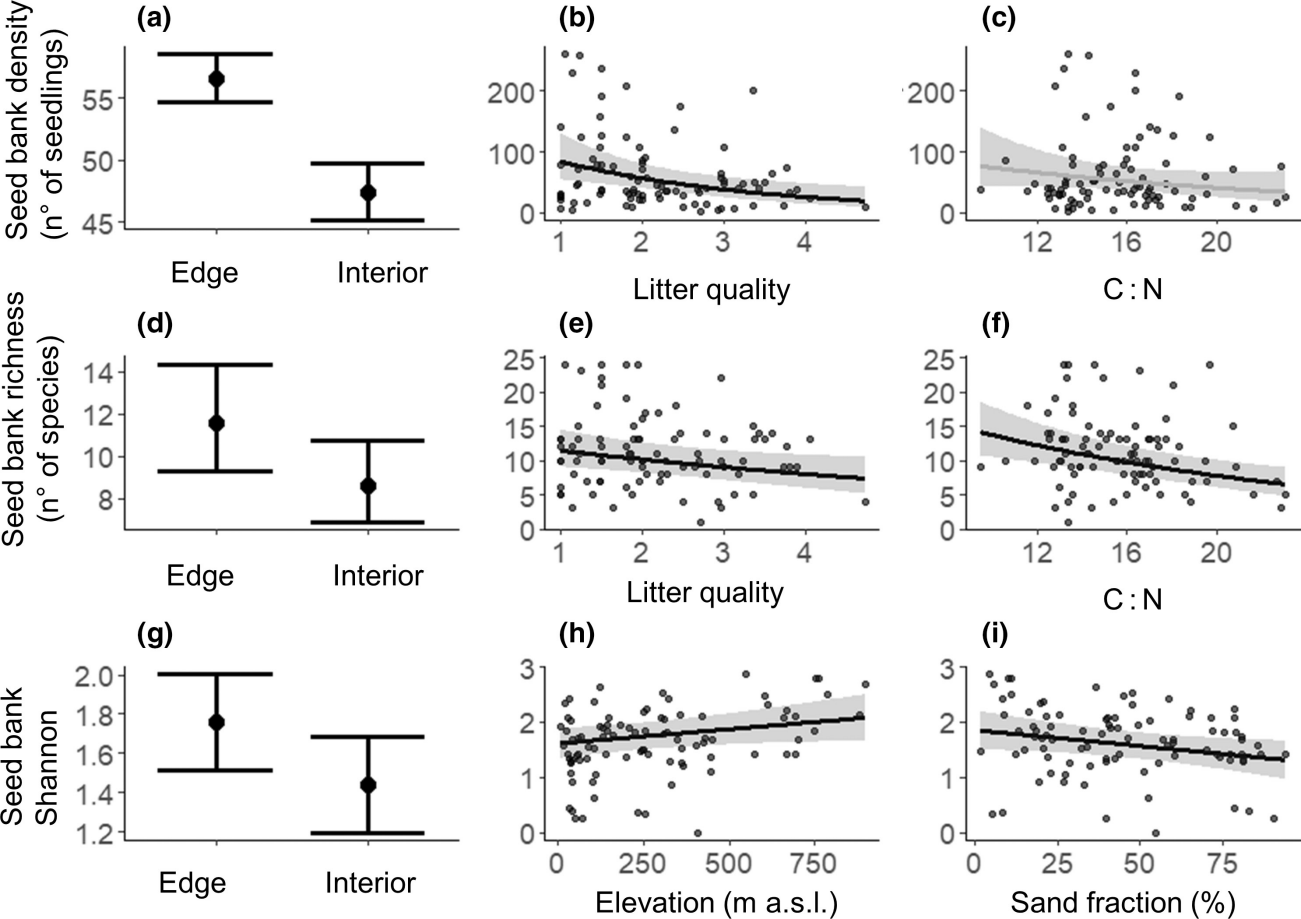
Seed bank density (a, b, c), species richness (d, e, f) and Shannon index (g, h, i) as a function of forest edge versus interior, elevation and environmental characteristics. Bars and lines show model predictions, respectively, of categorical and continuous variables. Black lines represent significant effects (*p* < .05), based on linear mixed models (LMMs) and generalized linear mixed models (GLMMs; Tables 1 and 2). Grey lines represent statistically non‐significant variables (c). Error bars (a, d, g) and grey ribbons (b, c, e, f, h, i) around the predicted point and lines, respectively, correspond to 95% confidence intervals. Model fit is shown in Tables 1 and 2.

Seed bank density, species richness and Shannon index were lower at the forest interior than in the edge (respectively *p* < .0001, *p* < .0001, *p* = .003, Figure 1). The forest edge soil seed bank hosted more seeds (66 and 59 seeds per plot on average, 1,381 and 1,232 seeds/m^2^, respectively) and was more species rich compared to the interior (13 and 10 species per plot on average, 270 and 208 species/m^2^, respectively).

Among the environmental variables, only soil pH, percentage of forest cover and PAI changed from the edge to the interior (Supporting Information Table S11). The soil pH was more acidic in the forest interior, whereas the percentage of forest cover and PAI were lower at the edge (Supporting Information Table S11). However, the seed bank density and the species richness were not affected directly by these variables. The seed bank density significantly declined with increasing litter quality (*p* = .002, Figure 1) whereas the species richness was negatively correlated with the C : N ratio and litter quality (respectively *p* < .0001 and *p* = .02, Figure 1). The Shannon index decreased with increasing content of sand (*p* = .04, Figure 1) and at lower elevation (*p* = .04, Figure 1).

### 3.2 Soil seed bank specialists and generalists

**FIGURE 2:**
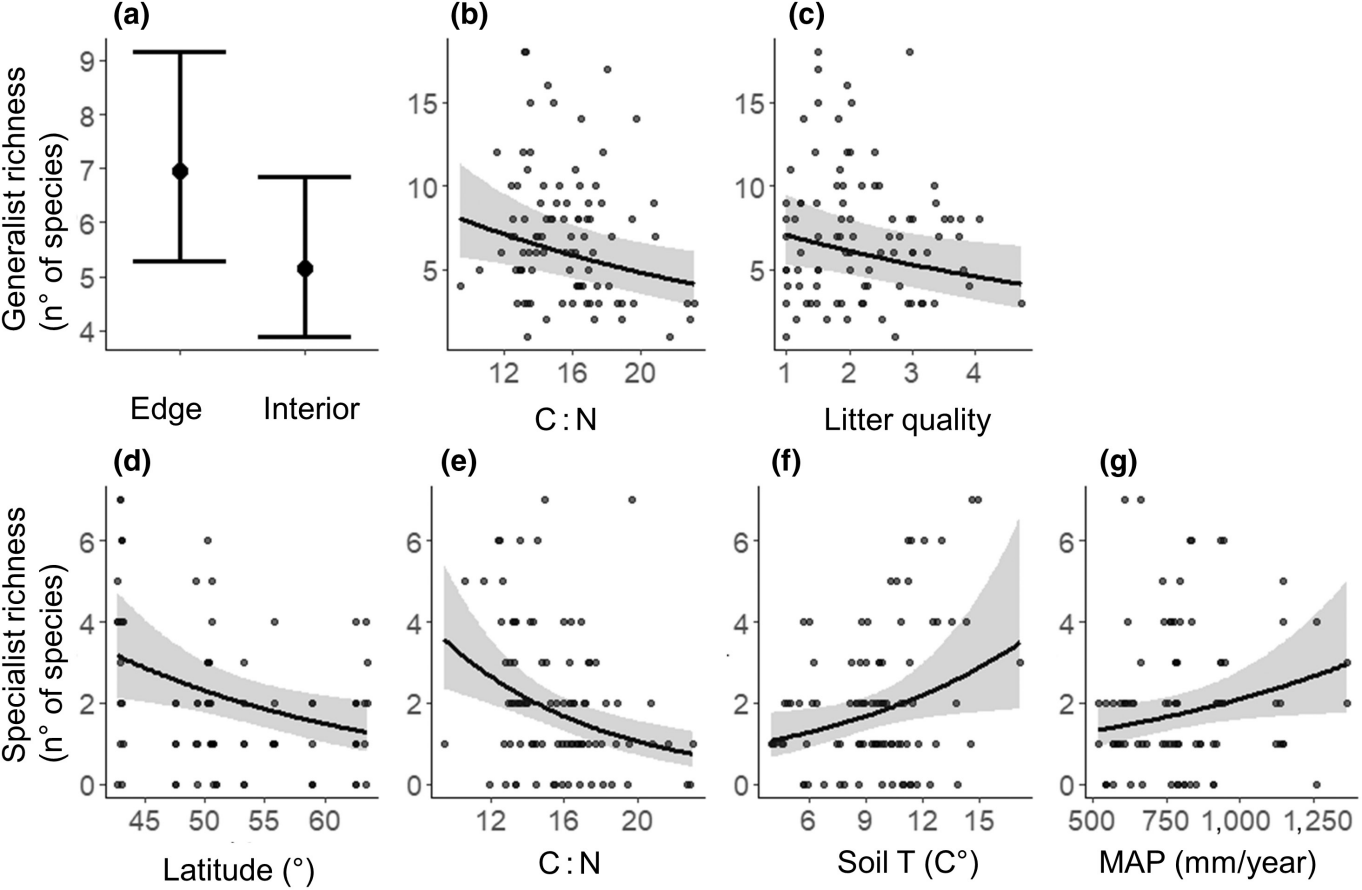
Generalist richness (a, b, c) and specialist richness (d, e, f, g) in the soil seed bank as a function of forest edge versus interior, latitude and environmental characteristics (C : N; litter quality; soil T = soil temperature and MAP = mean annual precipitation). Bars and lines show model predictions, respectively, of categorical and continuous variables. Only significant effects (*p* < .05), based on generalized linear mixed models (LMMs and GLMMs; Tables 1 and 2) are shown. Error bars (a) and grey ribbons (b–g) around the predicted point and lines, respectively, correspond to 95% confidence intervals. Model fit is shown in Tables 1 and 2.

Generalist species richness was lower in forest interiors compared to forest edges (*p* < .0001, Figure 2), while specialist richness and the proportion of forest specialists were not influenced by edge versus interior, but decreased towards northern latitudes (*p* = .008, Figure 2 and *p* = .005, Table 1, respectively).

**TABLE 1:** Summary of the results of generalized linear mixed models testing the influence of edge versus interior, latitude, elevation and forest type on the abundance and the diversity of the soil seed bank

	Edge versus interior	Latitude	Elevation	*R* ^2^ marginal	*R* ^2^ conditional
Seed bank density	−0.178***			.01	.26
Seed bank species richness	−0.295***			.10	.57
Seed bank/herb layer species richness	0.379*			.04	.28
Seed bank Shannon	−0.314**		0.124*	.11	.35
Seed bank evenness	0.021			.01	.08
Seed bank specialist richness	−0.272	−0.299**		.13	.17
Seed bank generalist richness	−0.304***			.09	.50
Seed bank proportion of specialist		−0.228**		.01	.01
Seed bank CWM temperature index	−0.207	−0.615**		.38	.62
Seed bank CWM seed mass			0.072	.03	.20
Seed bank CWM height		−0.504	−0.460***	.29	.61
Seed bank CWM SLA	−0.294	0.440	0.297*	.21	.45

*Notes*: Edge plot was used as reference category for edge versus interior. Values are parameter estimates after model selection, positive or negative values denote the direction of the effect. The significance of *p* values is given by *** for *p* < .001; ** for *p* < .01; * for *p* < .05. Variables not shown in the table were not included in any final model (forest type). Model fit was assessed based on marginal *R* ^2^, the proportion of variance explained by fixed effects and conditional *R* ^2^, the proportion of variance explained by both random and fixed effects (Nakagawa & Schielzeth, 2013). For explanation of the variables see Supporting Information Table S10.

Abbreviations: CWM, community weighted mean; SLA, specific leaf area.

Regarding the environmental predictors, absolute and relative specialist species richness increased with decreasing C : N (*p* < .001, Figure 2 and *p* = .006, Table 2, respectively), higher mean annual precipitation (MAP; *p* = .007, Figure 2 and *p* = .03, Table 2, respectively) and soil temperature (respectively, *p* = .02, Figure 2 and *p* = .01, Table 2). The total number of generalist species declined with increasing C : N ratio and litter quality (*p* = .06, *p* = .02, Figure 2, respectively).

**TABLE 2:** Summary of the results of generalized linear mixed models testing the influence of edaphic: (C : N, litter quality, % of sand and soil pH); climatic (MAP = mean annual precipitation and soil temperature) and landscape conditions (PAI = plant area index and % forest cover = proportion of forest in a radius of 500 m) on the abundance and the diversity of the soil seed bank

	C : N	Litter quality	% sand	Soil pH	Soil temperature	PAI	% forest cover	MAP	*R* ^2^ marginal	*R* ^2^ conditional
Seed bank density	−0.061	−0.347**							.10	.25
Seed bank species richness	−0.056***	−0.103*							.11	.57
Seed bank/herb layer species richness		−0.161				0.185*			.06	.25
Seed bank Shannon			−0.147*						.06	.33
Seed bank evenness				0.023			−0.001		.07	.08
Seed bank specialist richness	−0.116***				0.251*		0.009	0.186*	.27	.33
Seed bank generalist richness	−0.049**	−0.126*							.07	.52
Seed bank proportion of specialist	−0.095**				0.217*		0.010	0.247**	.06	.06
Seed bank CWM temperature index	−0.047			0.236*	0.584*		0.162		.50	.63
Seed bank CWM seed mass				0.254*		0.226*			.11	.11
Seed bank CWM height					0.459***				.22	.53
Seed bank CWM SLA				0.313**	−0.489*			0.172	.36	.45

*Notes*: Values are parameter estimates after model selection, positive or negative values denote the direction of the effect. The significance of *p* values is given by *** for *p* < .001; ** for *p* < .01; * for *p* < .05. Model fit was assessed based on marginal *R* ^2^, the proportion of variance explained by fixed effects and conditional *R* ^2^, the proportion of variance explained by both random and fixed effects (Nakagawa & Schielzeth, 2013). For explanation of the variables see Supporting Information Table S10.

Abbreviations: CWM, community weighted mean; SLA, specific leaf area.

### 3.3 Species composition

**FIGURE 3:**
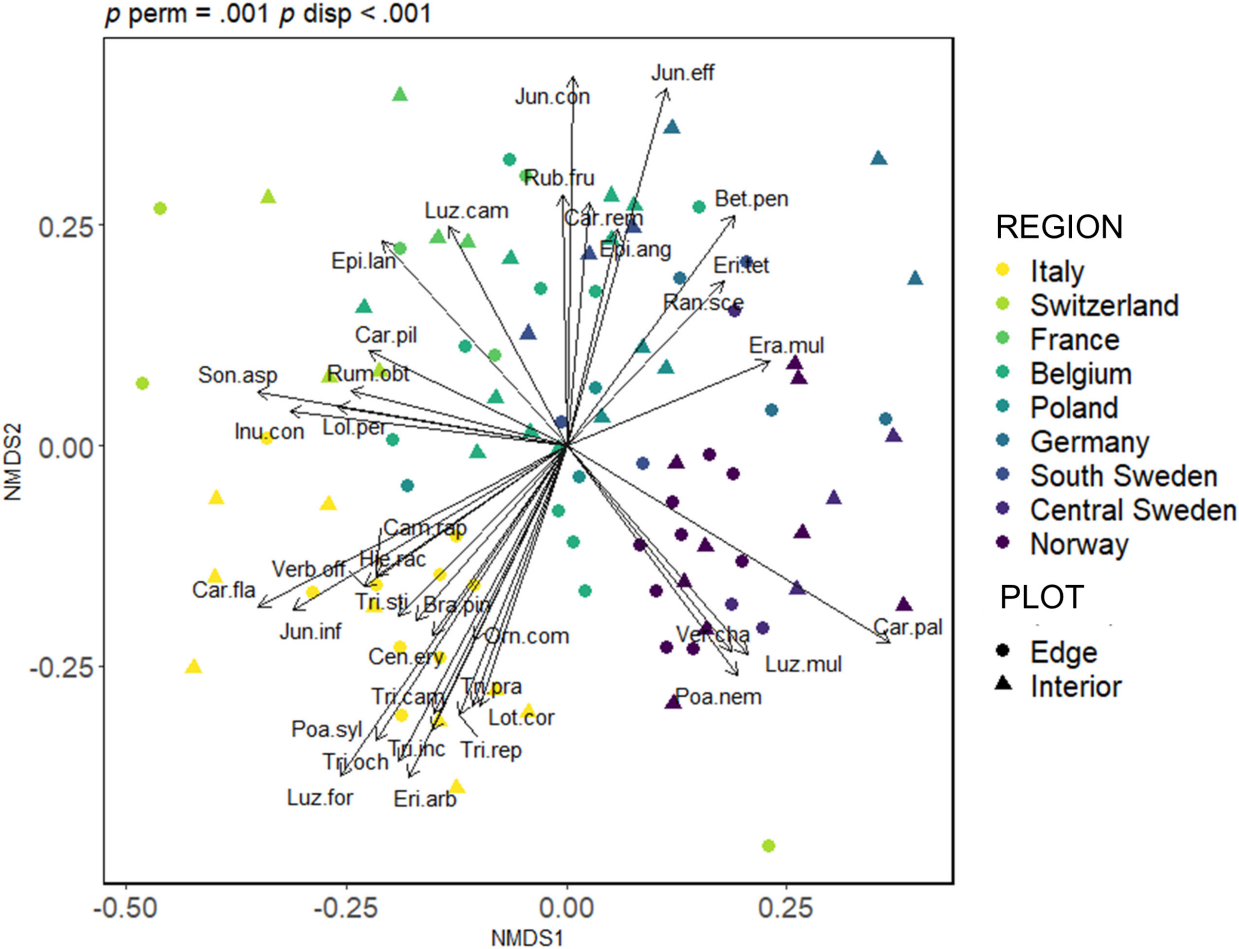
Non‐metric multidimensional scaling (NMDS) showing soil seed bank compositional dissimilarities between regions. Vectors represent significant species (*p* < .05). p perm: result of permutation test; p disp: result of the test for multivariate dispersion. The vector length is proportional to the correlation between ordination and the variable. Full species names can be found in Supporting Information Table S2.

The soil seed bank across regions was compositionally very variable (Figure 3). In particular, the soil seed bank in the southernmost versus northernmost sites in Italy and Norway differed significantly from those of the other regions. Moreover, the mean compositional Raup–Crick distance between edges and interiors was different between countries and was significantly higher in Switzerland (.93 ± .12) compared to France (.46 ± .25), central Sweden (.37 ± .32) and Norway (.48 ± .16) (respectively, *p* = .04, *p* < .01, *p* = .01). By contrast, this distance had more similar values for Italy (.61 ± .13), Belgium (.62 ± .19), Poland (.73 ± .08), Germany (.59 ± .17) and south Sweden (.64 ± 0.03).

### 3.4 Relationships between soil seed bank and herb layer

**FIGURE 4:**
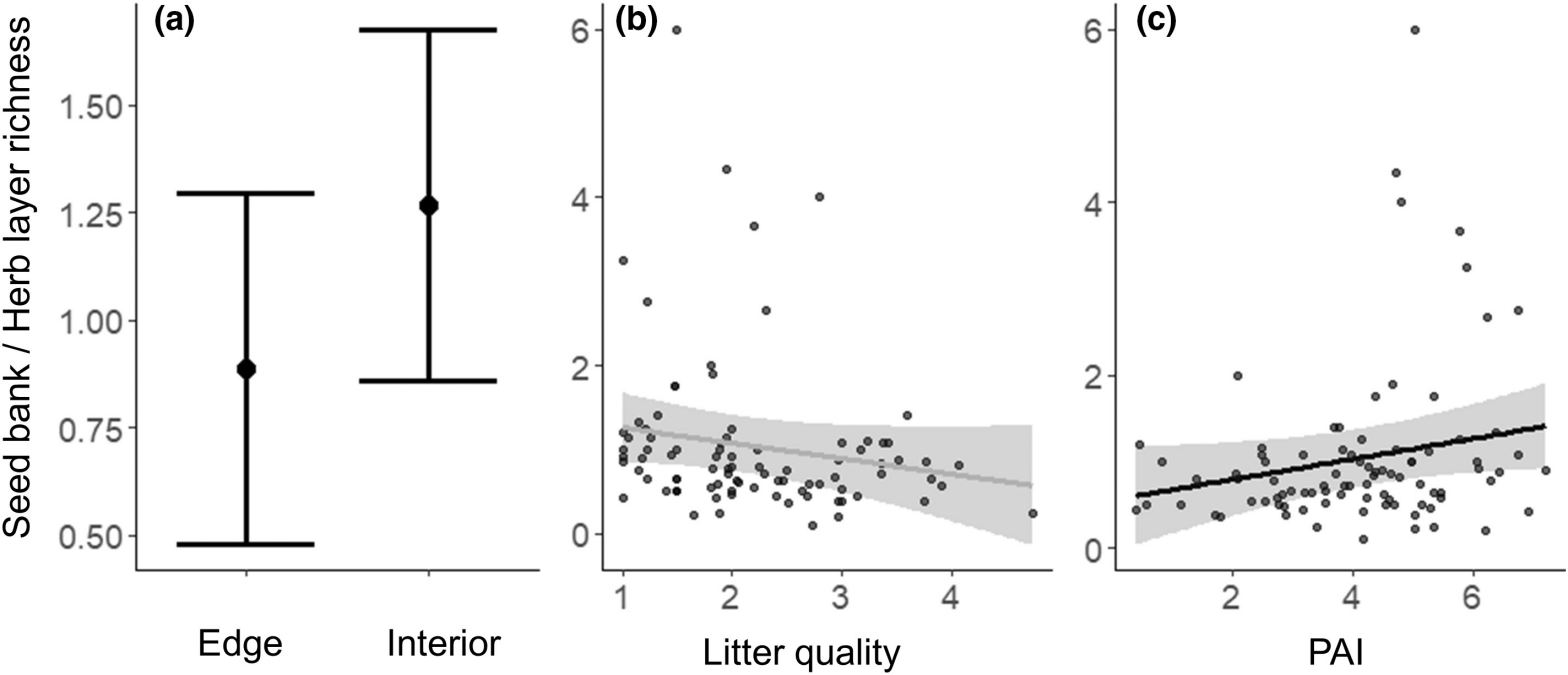
Ratio between soil seed bank and herb layer species richness as a function of forest edge versus interior plot (a), litter quality (b) and PAI = plant area index (c). A seed bank over herb layer richness > 1 indicates higher species richness in the soil seed bank. Bars and lines show model predictions, respectively, of categorical and continuous variables based on linear mixed models (Tables 1 and 2). Error bars (a) and grey ribbons (b, c) around the predicted point and lines, respectively, correspond to 95% confidence intervals. Grey line represents statistically non‐significant variables (b). Model fit is shown in Tables 1 and 2.

Vascular plant species richness values in the soil seed bank and in the herb layer were positively correlated (*p* < .0001, Spearman correlation was .52, Supporting Information Figure S4). The environment determined how species richness was partitioned across the herb layer and the soil seed bank. The proportion of species in the soil seed bank, compared to the herb layer, was higher at the forest interior than at the forest edge (*p* = .03; Figure 4). Indeed, the number of species in the soil seed bank became significantly larger than the number of species in the herb layer with increasing PAI (*p* = .05; Figure 4), reflecting the decrease in PAI from the forest interior to the edge (Supporting Information Table S11). The most abundant species recorded in both the soil seed bank and herb layer were *Deschampsia cespitosa*, *Rubus fruticosus* coll., *Poa nemoralis*, *Anthoxanthum odoratum*, *Calamagrostis epigejos*, *Brachypodium sylvaticum* and *Carex flacca*. On average, we recorded 12.9 ± 5.7 and 16.6 ± 7.8 species per plot in the soil seed bank and herb layer at the forest edges, respectively, versus 9.6 ± 4.1 and 12.5 ± 8.1 in the soil seed bank and herb layer of the forest interiors, respectively. Detailed analyses of the herb layer can be found in Supporting Information Tables S12 and S13.

### 3.5 Soil seed bank community characteristics

**FIGURE 5:**
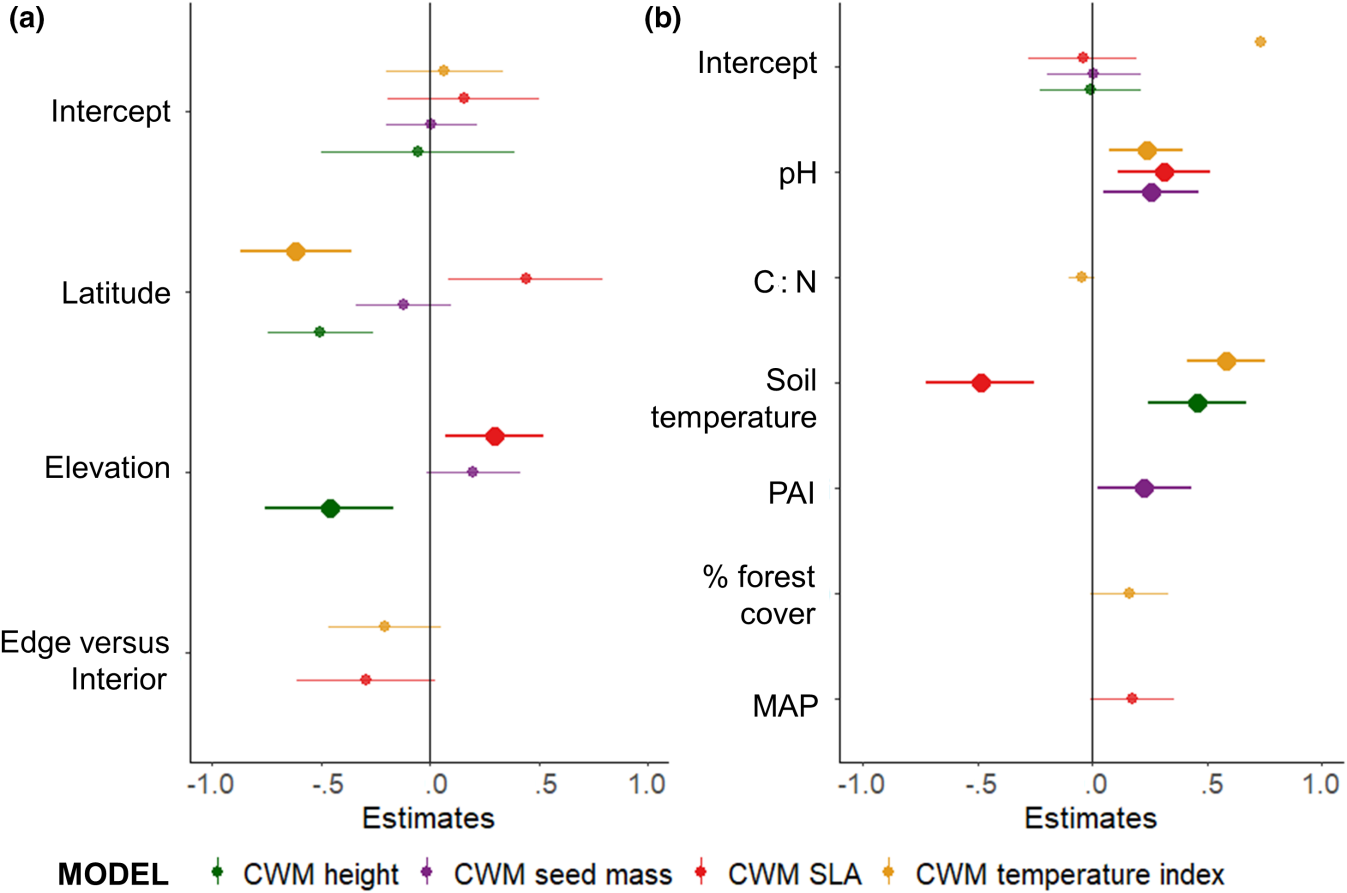
Estimates and 95% confidence intervals for the explanatory variables: (a) latitude, elevation, edge versus interior; (b) pH, C : N, (microclimatic) soil temperature, PAI = plant area index, % forest cover = proportion of forest in a radius of 500 m, MAP = mean annual precipitation; of linear mixed models for the community weighted mean (CWM) plant height, seed mass, specific leaf area (SLA) and temperature index of the soil seed bank. Edge plot was used as a reference category for the edge versus interior comparison. Significant variables are in bold. Variables that are not shown were not included in any final model; in (a) forest type; in (b) litter quality and % of sand. Marginal and conditional *R* ^2^ of the models are given in Tables 1 and 2.

The CWM values of plant height, seed mass, SLA and temperature index did not differ significantly between the forest edge and the interior but were related to latitude and elevation. As expected, the community temperature index of the soil seed bank decreased with increasing latitude (*p* = .006, Figure 5a). The CWM plant height decreased with elevation (*p* < .0001, Figure 5a), while the SLA increased (*p* = .01, Figure 5a).

Regarding the environmental variables, the community temperature index was positively influenced by soil pH (*p* = .01, Figure 5b) and the soil temperature (*p* = .01, Figure 5b). Seed mass was positively influenced by soil pH (*p* = .02, Figure 5b) and PAI (*p* = .03, Figure 5b). Plant height depended only on soil temperature, showing increasing values with higher temperatures (*p* < .001, Figure 5b). Finally, SLA was influenced by soil pH (positively, *p* = .005, Figure 5b) and soil temperature (negatively, *p* = .01, Figure 5b).

## 4 DISCUSSION

### 4.1 Forest edge versus interior soil seed bank

Our results support our hypothesis no. 1 and corroborate evidence from previous studies that found higher seed bank densities at temperate forest edges, with seed bank densities ranging between 4,171–20,831 seeds/m^2^ at the forest edge and 1,565–2,159 seeds/m^2^ at the interior and species richness of *c*. 16 and 2 species/m^2^, respectively (Cadenasso & Pickett, 2001; Devlaeminck et al., 2005; Honnay, Verheyen, & Hermy, 2002; Koncz et al., 2011). However, comparisons should be carefully made as the extension of the edge zone, the edge orientation and physiognomy, and the sampling depth and area are all main sources of variation (Honnay, Verheyen, & Hermy, 2002). As in the corresponding understorey vegetation (present work, Govaert et al., 2020; Pellissier et al., 2013), the lower density and species richness in the soil seed bank of forest interiors were mainly driven by a decrease in generalist species. By contrast, the specialist species richness in the soil seed bank remained constant between the forest edge and the forest interior. This is likely explained by changes in seed input from the aboveground vegetation (i.e. higher proportion of generalists in the herb layer of the edge) combined with higher seed longevity of generalist plants compared to forest specialists (Thompson et al., 1997). In the herb layer, generalist species are abundant in forest edge habitats, taking advantage of increased disturbance, warmer temperatures and more available resources (Brunet et al., 2011). At the edge, therefore, the current seed input could be of primary importance. Towards the interior, as seed input decreases (due to distance effects and dense forest edge zones that act as a barrier), the remnant seeds of the past vegetation (developed in different conditions due to canopy dynamics, forest management or past land use) could instead become relatively more abundant. These patterns would also explain the compositional differences between the edge versus interior that we found. Thus, forest edges hosted the seeds of many species (typical of fields or edges) that only in part penetrated towards the interior. At the same time, interiors were possibly richer in seeds of past vegetation that remained in the soil seed bank, thus causing this discrepancy.

Generalists produce a large amount of long‐lived seeds and have strong competitive and dispersal abilities (Büchi & Vuilleumier, 2014) whereas temperate forest herbs usually produce a few and often transient seeds and have present‐day restricted dispersal (Whigham, 2004). It is therefore commonly accepted that forest species reach lower seed bank densities than generalists (Plue, Colas, et al., 2017). Thus, the number of forest herbs in the soil seed bank found here was considerable, since 92% of the emerged seedlings were generalist plants (47 seeds per plot on average, 979.2 seeds/m^2^) while 8% were forest specialists (9 seeds per plot on average, 187.5 seeds/m^2^). This is within the broad range of variation reported so far for temperate forests (9–662 seeds/m^2^) (Chaideftou et al., 2011; Plue, De Frenne, et al., 2017). Our results support that generalist species are favoured at the ecotones between forest and open habitats and revealed that forest specialists are persisting and co‐existing with generalists in the soil seed bank of the forest edge, thus being able to overcome varying spatial and temporal conditions. While specialists generally succeed in their optimal habitats, generalists have broader environmental tolerance (Büchi & Vuilleumier, 2014). Usually, however, the lower light availability of the forest interior prevents the establishment of generalist edge species, despite the presence of their seeds in the soil (Pellissier et al., 2013). Generalists are thus likely to be outcompeted by specialists in most structured forest habitats, at least as long as no disturbance occurs. Yet, after strong disturbance events (i.e. large windthrow), microclimatic changes and edge effects could foster a rapid shift in plant community composition thanks to the soil seed bank.

### 4.2 Soil characteristics influencing the soil seed bank

Soil characteristics can have important effects on above and below‐ground community composition and diversity, and also affect the germination capacity of seeds (Heydari et al., 2013; Kůrová, 2016). Based on our results, differences in edaphic, climatic, structural and landscape characteristics between edges and interiors did not directly affect the soil seed bank in terms of density and species diversity. Forest edges differed from interiors in soil pH, percentage of forest cover in the surrounding landscape and PAI (Supporting Information Table S11). However, we found no influence of these variables on the density, species diversity and composition of the soil seed bank (Supporting Information Table S11). This could either mean that any one of these single factors can drive the responses of the seed bank in terms of density and species diversity, or that these are mainly driven by other factors that have not been considered in this study (e.g. light, air temperature instead of soil temperature, etc. etc.). Generally, our findings suggested that soil conditions (such as C : N and litter quality), rather than climatic or structural and landscape characteristics, have much stronger effects on the composition and diversity of the soil seed bank. Moreover, we showed that the soil seed bank and the corresponding herb layer diversity are influenced by different environmental drivers (litter quality, C : N ratio and the percentage of sand the former, soil pH and plant area index the latter) corroborating the evidence of Plue, De Frenne, et al. (2017) and Kůrová (2016) and calling for more integrated seed bank/herb layer analyses. Thick litter layers (low litter quality) on the forest floor may have posed a physical barrier for germination or could have inhibited seed germination through phytotoxic components (Facelli & Pickett, 1991), resulting in a higher number of seeds stored in the soil that emerged in the experiment after litter removal. These species were mainly generalist plants, suggesting that forest specialists may be better adapted to germinate under thick litter layers (Eriksson, 1995). Also macronutrients, such as nitrogen, can influence the emergence of seedlings from the soil seed bank (Thompson & Ooi, 2010) and exert a significant influence on the soil seed bank by improving the survival and longevity of many seeds in the soil (Bekker et al., 1998), which could explain why low C : N ratio in the soil seed bank was associated with higher species richness. Indeed, a low C : N ratio implies that soil organic matter is decomposing fast and that there is high net mineralization of nitrogen in the soil.

### 4.3 Soil seed bank variation with latitude and forest management

The latitudinal decline of forest specialists in the soil seed bank, observed also in the herb layer, could be related to a constraint in the growing season length in cooler temperate climates, which, among other effects, may limit the resource allocation to seed production in some species (Plue et al., 2013). Specialists may respond strongly to climatic factors compared to generalists as they have a higher proportion of species with limited seed longevity, which rely on regular yet small seed inputs to maintain a persistent seed bank (Whigham, 2004). Also, divergence among populations is known to be greatest in the south, where populations have persisted in local areas during both Quaternary glacials and interglacials (Petit et al., 2003). Due to species extinctions of the last ice age the species pools in the north would be still dominated by the relatively fewer species that have been able to disperse and recolonize widely and fast (Dynesius & Jansson, 2014). This, together with a multitude of other interacting factors, could eventually be responsible for lower compositional dissimilarities between the forest edge versus interior in northern regions. Higher or lower variation in soil seed bank composition between the edge versus interior could be related, for instance, to factors influencing the composition of the vegetation (and thus the composition of the seed rain), seed dispersal (e.g. predominance of species with zoochorous or anemochorous mechanisms), predation and former forest management or land use, that leave a persistent mark on the forest seed bank even after centuries of forest cover (Bossuyt & Hermy, 2001; Chambers & MacMahon, 1994; Jacquemyn et al., 2003). Interestingly, no different patterns in the soil seed bank composition and diversity emerged in relation to forest density. This is in contrast with evidence from the corresponding forest understorey, where generalists were more abundant in the interior of open rather than dense forests (Govaert et al., 2020). This could either mean that soil seed banks do not respond strongly to forest structure variations or that soil seed banks' responses lag behind compared to understorey vegetation, temporally buffering the pattern. The typical ecological profile of most forest plant species, especially their ability to form persistent populations through vegetative reproduction may be responsible for their slow response (Honnay, Verheyen, Butaye, et al., 2002). This would imply that what we currently observe today in the forest soil seed bank is not in equilibrium with the present landscape but rather reflects the past management. It is also possible that our study had some limitations in detecting a few species (most likely forest herbs) for which conditions for dormancy break and/or germination were not fully met. Unsuitable germination conditions may result in an underrepresentation of the habitat specialists in the soil seed bank and thus confound the results (Plue, Colas, et al., 2017).

### 4.4 Relationship between soil seed bank and herb layer species richness

The species richness values of the seed bank and the herb layer were positively related and depended on microclimate, thus supporting our hypothesis no. 3. Our results are in line with previous findings that species transition between the soil seed bank and the herb layer is driven as a function of the environment, mainly of light availability (Plue, De Frenne, et al., 2017; Royo & Ristau, 2013). In the forest interior, canopy closure does not favour the germination of light‐dependent species that are maintained in their dormant seed bank state (Plue et al., 2010), increasing the number of species occurring in the soil seed bank. Conversely, the higher light availability of the forest edge enables the establishment of heliophilous species from the soil seed bank in the herb layer, increasing their number. At the edge, therefore, the soil seed bank can better express itself in the vegetation community, also due to the steep microclimatic gradient and less stable conditions over time than in the forest interior. Forest herbs and light‐dependent species from the soil seed bank co‐exist in space competing with each other and filling their separated niche (Plue, De Frenne, et al., 2017). Any changes in forest or canopy cover (through natural canopy dynamics, forest management or forest fragmentation) create a heterogeneous understorey environment in terms of light availability (De Frenne et al., 2010; Meeussen et al., 2021), soil nutrients (Fraterrigo et al., 2005) and soil moisture (James et al., 2003) that drive continuous herb layer–seed bank interactions.

### 4.5 Soil seed bank community traits

Relatively few studies have hitherto explored functional traits of species in seed banks, although these can reveal less obvious but equally meaningful results as species life‐history or ecological groups (Larson & Suding, 2022; Medeiros‐Sarmento et al., 2021; Pakeman & Eastwood, 2013). Here, the seed bank communities at the edge versus interior unexpectedly expressed a convergent profile in the traits here considered, despite their divergence in species composition. Hence, the present data do not support our fourth hypothesis. This suggests that the similar functional convergence observed in the realized herb layer (present work, De Pauw et al., 2021) is likely not the result of a microclimatic filter (edge versus interior) but is ‘inherent’, that is, the same trait profile is already present in the stored community, the soil seed bank. Hence, the general trait profile seems largely independent from the species composition of the soil seed bank, possibly due to the presence of more species with similar traits. Overall, our data suggest that the edge and interior communities may contribute similarly to ecological processes and display similar functional responses to changes, though this remains to be tested including a wider range of traits. The trait‐based approach also provided complementary information about forest soil seed bank communities along the gradients. For instance, soil pH and PAI acted as filtering factors on traits related to dispersal and light requirements. Moreover, we observed different effects of the environmental variables in trait selection between the aboveground vegetation and corresponding seed bank communities, corroborating the findings of Larson and Suding (2022). In the understorey, for instance, the CWM SLA increased with latitude and in dense forests (Supporting Information Table S12), whereas it depended more on elevation in the soil seed bank (Figure 5). This finding not only confirmed that plant resource acquisition strategies are linked to seed bank patterning, but also provided insights into the potential role of the functional composition of the stored community if conditions change. Our findings support previous evidence that species with larger SLA are better adapted to longer and colder winters, ameliorate resource acquisition and overcome the constraints of a shorter growing season (Gonzalo‐Turpin & Hazard, 2009). Additionally, smaller plant size at the highest elevation is an important adaptation strategy that is commonly assumed to provide several advantages including warmer microclimatic conditions close to the ground and protection from wind (Happonen et al., 2021). Our study also supports the hypothesis that soil seed banks of dense forests are composed of larger seeds (Tautenhahn et al., 2008) and could thus tolerate a higher degree of stress in the form of shade (Moles et al., 2005).

## 5 CONCLUSIONS

We detected significant differences in forest edge versus interior soil seed banks in ancient deciduous forests of Europe, as well as large variation in species composition across the latitudinal gradient. Our results underpin how forest edge effects alter the soil seed bank diversity and composition, increasing the proportion of generalist species and thus potentially fostering a rapid shift in plant community composition depending on environmental conditions. This study also demonstrates that many typical forest species are part of the soil seed bank, underlining the importance of the soil seed bank for forest understorey species and showing that edge microhabitat is not unsuitable for their persistence in the form of seeds. Hence, seed banks may support greater resilience than expected in our fragmented forest ecosystems, at least in the short term. Using a novel trait‐based approach, we detected the same edge versus interior functional convergence that occurs in the aboveground vegetation, suggesting no microclimate filtering effect and that plant resource acquisition strategies are linked to soil seed bank patterning. This can provide significant insights on how stored functional composition could impact vegetation under environmental change.

## AUTHOR CONTRIBUTIONS

Pieter De Frenne, Federico Selvi, Jan Plue and Cristina Gasperini conceptualized and designed the research. Cristina Gasperini performed the data analyses and wrote the original draft. All authors collected data and contributed substantially to revisions.

## CONFLICT OF INTEREST

The authors declare that they have no known competing financial interests or personal relationships that could have appeared to influence the work reported in this paper.

## BIOSKETCH

**Cristina Gasperini** is a PhD student interested in the effects of climate warming and habitat fragmentation on forest soil seed banks. Her research frames within the FORMICA research project (http://www.formica.ugent.be/).

## Supporting information


Supinfo S1

Supinfo S2

## Data Availability

The data that support the findings of this study are openly available in Figshare at https://doi.org/10.6084/m9.figshare.17151902.
