# Supplementary material for: Soil seed bank responses to edge effects in temperate European forests
Source: Glob Ecol Biogeogr. 2022 Jul 16;31(9):1877–93. doi: 10.1111/geb.13568 (PMC9546374; doi:10.1111/geb.13568)
Supplement: Supplementary file 1 — Supinfo S1 [file GEB-31-1877-s001.docx]

Appendix A: Supplementary materials


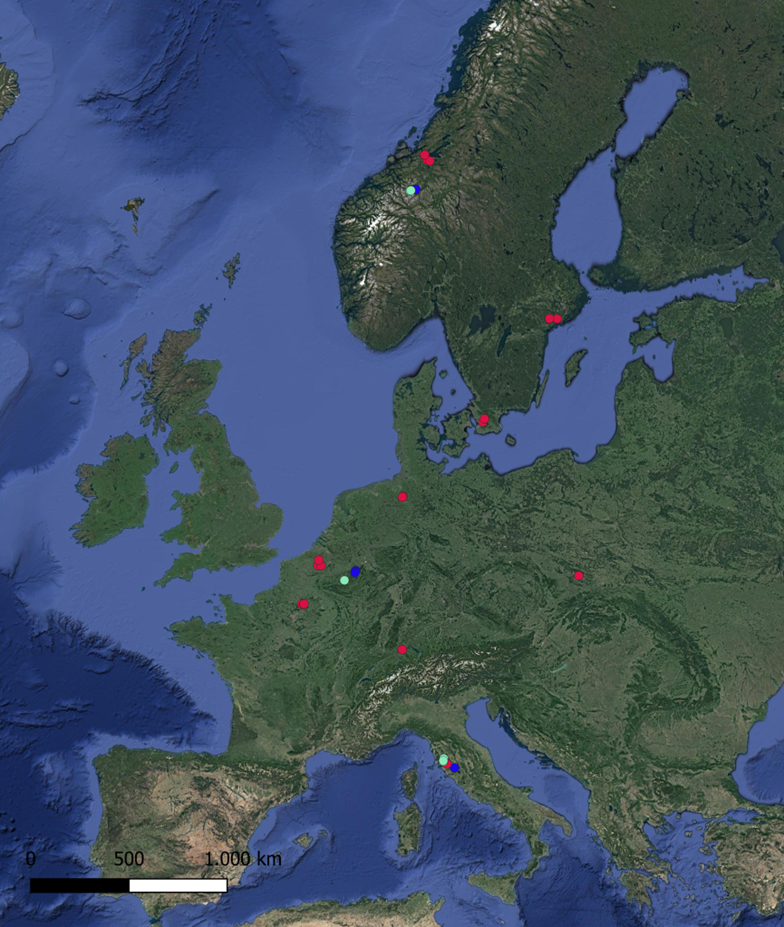


**Figure A1.** Map showing the 15 sites involved in seed bank sampling along a latitudinal gradient. The nine lowland regions are represented by red dots, from south to north these are located in Italy, Switzerland, France, Belgium, Poland, Germany, Southern Sweden, Central Sweden and Norway. Blue and light blue dots show medium and high elevational sites in Italy, Belgium and Norway.

**Table A1**: Site descriptions including region, elevation and forest structural types. Precipitation (MAP=mean annual precipitation) and temperature data (MAT= mean annual temperature) were obtained from CHELSA version 1.2 and extracted for each site (n=45) (mean of the period 1979-2013 at a horizontal resolution of 30 arc sec) (Karger et al., 2017). For an extensive description of site selection and measurements see Meeussen et al. (2020) and Govaert et al. (2020). Nomenclature follows Euro+Med (2006).

| **Region** | **Elevational level** | **Forest structural type** | **Lat (°N)** | **Long (°E)** | **Mean elevation (m)** | **Tree cover (%)** | **Shrub cover (%)** | **Basal area (m2/ha)** | **MAT (°C)** | **MAP (mm year-1)** | **Dominant tree** | **Dominant shrub species** |
| --- | --- | --- | --- | --- | --- | --- | --- | --- | --- | --- | --- | --- |
| Italy | Low | Dense | 42.97 | 11.21 | 133 | 87.5 | 29 | 21.03935 | 15.3 | 620 | Quercus cerris | Ligustrum vulgare,  Crataegus monogyna,  Quercus cerris |
| Italy | Low | Intermediate | 42.95 | 11.22 | 189 | 82.5 | 39.5 | 17.78324 | 15.1 | 665 | Quercus cerris | Arbutus unedo,  Erica arborea |
| Italy | Low | Open | 42.95 | 11.23 | 126 | 67.5 | 0 | 12.21914 | 15.4 | 610 | Quercus cerris | / |
| Italy | Medium | Dense | 43.18 | 11.04 | 423 | 76 | 12.5 | 20.15934 | 13.2 | 762 | Quercus cerris | Acer campestre,  Quercus cerris,  Cornus sanguinea |
| Italy | Medium | Intermediate | 43.05 | 11.01 | 619 | 72.5 | 7.5 | 17.39738 | 12.6 | 834 | Quercus cerris | / |
| Italy | Medium | Open | 43.09 | 11.01 | 558 | 57.5 | 34.5 | 5.542438 | 12.6 | 829 | Quercus cerris | Fraxinus ornus,  Quercus cerris,  Prunus avium |
| Italy | High | Dense | 42.78 | 11.66 | 779 | 87.5 | 29.5 | 27.43982 | 10.8 | 794 | Quercus cerris,  Ostrya carpinifolia,  Prunus avium | Fraxinus ornus,  Acer pseudoplatanus,  Quercus cerris |
| Italy | High | Intermediate | 43.1 | 10.98 | 891 | 95 | 5 | 30.88117 | 10.7 | 942 | Quercus cerris,  Ostrya carpinifolia,  Acer monspessulanum | Fraxinus ornus,  Acer monspessulanum,  Juniperus communis |
| Italy | High | Open | 42.79 | 11.66 | 753 | 90 | 3.75 | 20.43627 | 11.6 | 768 | Quercus cerris | / |
| Switzerland | Low | Dense | 47.6 | 8.63 | 418 | 125 | 41 | 35.02701 | 10.46 | 910 | Fagus sylvatica,  Tilia cordata,  Quercus petraea | Fagus sylvatica,  Carpinus betulus,  Acer pseudoplatanus |
| Switzerland | Low | Intermediate | 47.6 | 8.63 | 419 | 97 | 40 | 39.76312 | 10.46 | 910 | Fagus sylvatica,  Quercus petraea | Fagus sylvatica,  Picea abies |
| Switzerland | Low | Open | 47.6 | 8.64 | 429 | 117.5 | 30 | 37.79167 | 10.46 | 911 | Fagus sylvatica,  Tilia cordata | Fagus sylvatica,  Tilia cordata |
| France | Low | Dense | 49.34 | 2.82 | 56 | 50.25 | 12.5 | 22.6412 | 11.02 | 634 | Fagus sylvatica,  Quercus robur,  Carpinus betulus | Carpinus betulus,  Fagus sylvatica |
| France | Low | Intermediate | 49.32 | 2.93 | 150 | 85 | 33.25 | 28.25193 | 10.5 | 739 | Carpinus betulus,  Quercus robur,  Acer campestre | Fagus sylvatica,  Crataegus monogyna,  Corylus avellana |
| France | Low | Open | 49.35 | 2.96 | 131 | 0.5 | 0 | 11.69753 | 10.6 | 671 | Quercus petraea,  Fagus sylvatica | Fagus sylvatic |
| Poland | Low | Dense | 50.4 | 18.85 | 316 | 58.5 | 2.75 | 19.12693 | 8.2 | 782 | Quercus robur,  Fagus sylvatica | Fagus sylvatica,  Cornus sanguinea,  Tilia cordata |
| Poland | Low | Intermediate | 50.4 | 18.84 | 310 | 117.75 | 22.75 | 35.0625 | 8.2 | 782 | Acer pseudoplatanus,  Tilia cordata,  Quercus rubra | Tilia cordata,  Fagus sylvatica,  Acer pseudoplatanus |
| Poland | Low | Open | 50.4 | 18.85 | 323 | 97.5 | 0.5 | 15.15201 | 8.22 | 777 | Quercus robur,  Fagus sylvatica,  Quercus rubra | Rhamnus frangula,  Fagus sylvatica,  Acer pseudoplatanus |
| Belgium | Low | Dense | 50.77 | 3.75 | 106 | 110 | 26 | 37.09452 | 10.1 | 848 | Quercus robur,  agus sylvatica,  Acer pseudoplatanus | Corylus avellana,  Carpinus betulus,  Fraxinus excelsior |
| Belgium | Low | Intermediate | 50.77 | 3.93 | 67 | 100 | 16.5 | 46.43287 | 10.4 | 863 | Quercus robur,  Populus canadensis,  Acer pseudoplatanus | Corylus avellana,  Sambucus nigra |
| Belgium | Low | Open | 50.97 | 3.8 | 31 | 87.5 | 70 | 8.112654 | 10.5 | 809 | Fagus sylvatica,  Quercus robur,  Quercus rubra | Quercus rubra,  Corylus avellana,  Tilia cordata |
| Belgium | Medium | Dense | 50.23 | 5.28 | 243 | 70 | 30 | 25.20293 | 9.5 | 937 | Carpinus betulus,  Quercus petraea,  Alnus glutinosa | Carpinus betulus |
| Belgium | Medium | Intermediate | 50.23 | 5.29 | 247 | 92.5 | 30 | 21.61921 | 9.5 | 934 | Quercus petraea,  Carpinus betulus | Carpinus betulus |
| Belgium | Medium | Open | 50.23 | 5.29 | 246 | 82.5 | 2.5 | 31.99807 | 9.5 | 934 | Quercus petraea,  Carpinus betulus | Carpinus betulus |
| Belgium | High | Dense | 50.58 | 5.94 | 351 | 92.5 | 35 | 43.56096 | 8.6 | 1143 | Fagus sylvatica,  Quercus petraea,  Carpinus betulus | Corylus avellana,  Ilex aquifolium,  Fagus sylvatica |
| Belgium | High | Intermediate | 50.58 | 5.94 | 360 | 81.5 | 68 | 23.04938 | 8.6 | 1143 | Quercus petraea,  Carpinus betulus | Corylus avellana,  Acer pseudoplatanus,  Sorbus aucuparia |
| Belgium | High | Open | 50.52 | 5.89 | 340 | 55 | 16 | 24.28861 | 8.8 | 1120 | Quercus petraea | Quercus petraea,  Corylus avellana,  Fagus sylvatica |
| Germany | Low | Dense | 53.24 | 8.67 | 40 | 65.25 | 67.5 | 19.74036 | 9.3 | 792 | Quercus robur,  Betula pendula | Sorbus aucuparia,  Prunus serotine,  Ilex aquifolium |
| Germany | Low | Intermediate | 53.21 | 8.65 | 42 | 87.5 | 24.5 | 45.33951 | 9.4 | 788 | Fagus sylvatica,  Quercus robur,  Carpinus betulus | Fagus sylvatica |
| Germany | Low | Open | 53.21 | 8.64 | 38 | 147.75 | 46.5 | 37.20139 | 9.4 | 788 | Fagus sylvatica,  Quercus robur,  Betula pendula | Fagus sylvatica,  Ilex aquifolium,  Carpinus betulus |
| Southern Sweden | Low | Dense | 55.73 | 13.3 | 81 | 65 | 85.25 | 41.15162 | 7.72 | 677 | Quercus robur | Corylus avellana,  Sorbus aucuparia,  Crataegus monogyna |
| Southern Sweden | Low | Intermediate | 55.83 | 13.41 | 104 | 92.5 | 10 | 49.24691 | 7.5 | 749 | Fagus sylvatica,  Quercus robur | Fagus sylvatica |
| Southern Sweden | Low | Open | 55.84 | 13.4 | 117 | 87.5 | 5.5 | 26.74846 | 7.4 | 778 | Quercus robur,  Fagus sylvatica | Fagus sylvatica,  Carpinus betulus,  Corylus avellana |
| Central Sweden | Low | Dense | 58.95 | 17.61 | 14 | 87.5 | 65 | 46.37809 | 6.8 | 519 | Quercus robur,  Tilia cordata | Corylus avellana,  Tilia cordata |
| Central Sweden | Low | Intermediate | 58.96 | 17.15 | 43 | 76.5 | 48.5 | 25.12346 | 6.7 | 547 | Populus tremula | Corylus avellana,  Picea abies |
| Central Sweden | Low | Open | 58.96 | 17.15 | 43 | 92.5 | 0 | 20.74846 | 6.7 | 546 | Betula pendula,  Populus tremula,  Tilia cordata | Populus tremula |
| Norway | Low | Dense | 63.37 | 10.09 | 35 | 99 | 30 | 39.70448 | 6.1 | 1258 | Alnus incana,  Ulmus glabra,  Salix caprea | Prunus padus,  Sorbus aucuparia,  Alnus incana |
| Norway | Low | Intermediate | 63.35 | 10.23 | 42 | 130 | 20 | 36.11154 | 6.1 | 1136 | Ulmus glabra,  Alnus incana | Ulmus glabra,  Prunus padus,  Alnus incana |
| Norway | Low | Open | 63.5 | 9.93 | 27 | 22 | 69 | 14.45525 | 6.2 | 1361 | Populus tremula | Corylus avellana,  Betula pendula,  Picea abies |
| Norway | Medium | Dense | 62.58 | 9.14 | 240 | 107.5 | 37.75 | 35.51852 | 3.34 | 697 | Ulmus glabra,  lnus incana,  Prunus padus | Prunus padus,  Alnus incana,  Salix caprea |
| Norway | Medium | Intermediate | 62.56 | 9.08 | 221 | 91.5 | 36.5 | 30.17978 | 4.9 | 596 | Alnus incana,  Salix caprea | Salix caprea,  Alnus incana |
| Norway | Medium | Open | 62.57 | 9.13 | 283 | 70 | 85 | 30.16049 | 4.4 | 612 | Betula pubescens | Corylus avellana,  Betula pubescens |
| Norway | High | Dense | 62.61 | 9.43 | 676 | 65 | 50 | 17.68171 | 2.4 | 572 | Betula pubescens | Betula pubescens,  Alnus incana,  Sorbus aucuparia |
| Norway | High | Intermediate | 62.59 | 9.39 | 675 | 34.5 | 5.5 | 17.46557 | 2.8 | 586 | Betula pubescens | Betula pubescens,  Alnus incana |
| Norway | High | Open | 62.6 | 9.43 | 681 | 62.5 | 35 | 15.40741 | 2.4 | 572 | Betula pubescens | Betula pubescens |

**Table A2**. Complete list of species found in the herb layer and the soil seed bank, at the forest edge and interior.

| code | Species | SEED BANK |  | HERB LAYER |  |
| --- | --- | --- | --- | --- | --- |
|  |  | Edge | Interior | Edge | Interior |
| Ace.cam | *Acer campestre* |  |  | ✓ | ✓ |
| Ace.mon | *Acer monspessulanum* |  |  | ✓ |  |
| Ace.pla | *Acer platanoides* |  |  | ✓ | ✓ |
| Ace.pse | *Acer pseudoplatanus* |  |  | ✓ | ✓ |
| Alc.mill | *Achillea millefolium* |  |  | ✓ |  |
| Aco.lyc | *Aconitum lycoctonum ssp. septentrionale* |  |  | ✓ | ✓ |
| Act.spi | *Actaea spicata* |  |  |  | ✓ |
| Aeg.pur | *Aegonychon purpurocaeruleum* | ✓ |  | ✓ | ✓ |
| Aeg.pod | *Aegopodium podagraria* |  |  | ✓ | ✓ |
| Agr.eup | *Agrimonia eupatoria* |  |  | ✓ |  |
| Agr.can | *Agrostis canina* | ✓ | ✓ |  |  |
| Agr.cap | *Agrostis capillaris* | ✓ | ✓ | ✓ |  |
| Agr.mer | *Agrostis mertensii* |  |  | ✓ |  |
| Agr.spe1 | *Agrostis spec. 1* |  |  | ✓ | ✓ |
| Agr.spe2 | *Agrostis spec. 2* |  |  | ✓ | ✓ |
| Aju.pir | *Ajuga pyramidalis* | ✓ |  |  | ✓ |
| Aju.rep | *Ajuga reptans* | ✓ |  |  | ✓ |
| Alc.alp | *Alchemilla alpina* |  |  | ✓ |  |
| Alc.spe | *Alchemilla spec.* |  |  | ✓ |  |
| All.pet | *Alliaria petiolata* |  | ✓ | ✓ |  |
| All.pen | *Allium pendulinum* |  |  | ✓ | ✓ |
| Aln.inc | *Alnus incana* | ✓ | ✓ | ✓ | ✓ |
| Ana.arv | *Anagallis arvensis* | ✓ | ✓ |  |  |
| Ane.app | *Anemone apennina* |  |  | ✓ | ✓ |
| Ane.nem | *Anemone nemorosa* |  |  | ✓ | ✓ |
| Ani.ste | *Anisantha sterilis* | ✓ | ✓ |  |  |
| Ant.lil | *Anthericum liliago* |  |  |  | ✓ |
| Ant.odo | *Anthoxanthum odoratum* | ✓ | ✓ | ✓ | ✓ |
| Ant.syl | *Anthriscus sylvestris* | ✓ |  | ✓ |  |
| Aph.arv | *Aphanes arvensis* |  | ✓ |  |  |
| Ara.tha | *Arabidopsis thaliana* |  | ✓ |  |  |
| Are.ser | *Arenaria serpyllifolia* | ✓ | ✓ |  |  |
| Ari.lut | *Aristolochia lutea* |  |  | ✓ | ✓ |
| Arr.ela | *Arrhenatherum elatius subsp. elatius* |  |  | ✓ | ✓ |
| Asa.eur | *Asarum europaeum* |  |  | ✓ |  |
| Asp.acu | *Asparagus acutifolius* |  |  | ✓ | ✓ |
| Asp.adi | *Asplenium adiantum-nigrum subsp. Onopteris* |  |  | ✓ | ✓ |
| Ast.spe | *Asteraceae spec.* |  |  | ✓ |  |
| Ast.gly | *Astragalus glycyphyllos* |  | ✓ |  |  |
| Ath.fil | *Athyrium filix-femina* |  |  | ✓ | ✓ |
| Ave.ste | *Avena sterilis* |  | ✓ |  |  |
| Ave.fle | *Avenella flexuosa* |  |  | ✓ | ✓ |
| Bar.vul | *Barbarea vulgaris* | ✓ | ✓ |  |  |
| Bel.per | *Bellis perennis* | ✓ | ✓ |  |  |
| Bet.pen | *Betula pendula* | ✓ | ✓ |  |  |
| Bet.pub | *Betula pubescens* | ✓ | ✓ | ✓ | ✓ |
| Bra.pin | *Brachypodium pinnatum* | ✓ | ✓ | ✓ | ✓ |
| Bra.syl | *Brachypodium sylvaticum* | ✓ | ✓ | ✓ | ✓ |
| Bro.hor | *Bromus hordeaceus* | ✓ |  |  |  |
| Bro.ram | *Bromus ramosus* |  | ✓ | ✓ |  |
| Bro.spe | *Bromus sp.* | ✓ |  |  |  |
| Bud.dav | *Buddleja davidii* |  | ✓ |  |  |
| Cal.epi | *Calamagrostis epigejos* | ✓ | ✓ | ✓ | ✓ |
| Cal.sta | *Callitriche stagnalis* |  | ✓ |  |  |
| Cal.vul | *Calluna vulgaris* | ✓ | ✓ | ✓ |  |
| Cal.sep | *Calystegia sepium* |  |  | ✓ |  |
| Cam.lat | *Campanula latifolia* |  |  | ✓ |  |
| Cam.rap | *Campanula rapunculus* |  | ✓ | ✓ |  |
| Cam.rot | *Campanula rotundifolia* | ✓ |  | ✓ |  |
| Crd.hir | *Cardamine hirsuta* |  |  |  | ✓ |
| Car.pra | *Cardamine pratensis* | ✓ |  |  |  |
| Car.dig | *Carex digitata* |  | ✓ | ✓ |  |
| Car.dis | *Carex distachya* |  |  | ✓ | ✓ |
| Car.div | *Carex divulsa* |  |  |  | ✓ |
| Car.fla | *Carex flacca* | ✓ | ✓ | ✓ | ✓ |
| Car.hir | *Carex hirta* | ✓ | ✓ | ✓ |  |
| Car.olb | *Carex olbiensis* | ✓ |  | ✓ | ✓ |
| Car.ova | *Carex ovalis* | ✓ |  |  |  |
| Car.pal | *Carex pallescens* | ✓ | ✓ | ✓ | ✓ |
| Car.pen | *Carex pendula* |  | ✓ |  |  |
| Car.pil | *Carex pilosa* | ✓ | ✓ | ✓ | ✓ |
| Car.pilu | *Carex pilulifera* | ✓ | ✓ | ✓ | ✓ |
| Car.rem | *Carex remota* | ✓ | ✓ |  | ✓ |
| Car.spe | *Carex spec.* | ✓ |  | ✓ |  |
| Car.syl | *Carex sylvatica* | ✓ | ✓ | ✓ | ✓ |
| Car.bet | *Carpinus betulus* |  |  | ✓ | ✓ |
| Cas.sat | *Castanea sativa* |  |  | ✓ |  |
| Cen.jac | *Centaurea jacea subsp. Gaudinii* |  |  | ✓ |  |
| Cen.ery | *Centaurium erythraea* | ✓ | ✓ |  |  |
| Cep.lon | *Cephalanthera longifolia* |  |  |  | ✓ |
| Cer.arv | *Cerastium arvense* | ✓ |  | ✓ | ✓ |
| Cer.fon | *Cerastium fontanum* | ✓ |  |  |  |
| Cer.glo | *Cerastium glomeratum* | ✓ | ✓ |  |  |
| Cer.sem | *Cerastium semidecandrum* |  | ✓ |  |  |
| Cer.cla | *Ceratocapnos claviculata* |  |  | ✓ | ✓ |
| Cha.tem | *Chaerophyllum temulum* | ✓ | ✓ |  |  |
| Che.alb | *Chenopodium album* | ✓ | ✓ |  |  |
| Cic.int | *Cichorium intybus* | ✓ |  |  |  |
| Cir.lut | *Circaea lutetiana* | ✓ |  | ✓ |  |
| Cir.arv | *Cirsium arvense* | ✓ | ✓ | ✓ |  |
| Cis.sal | *Cistus salviifolius* | ✓ | ✓ | ✓ | ✓ |
| Cis.spe | *Cistus sp.* | ✓ |  |  |  |
| Cle.vit | *Clematis vitalba* |  | ✓ | ✓ | ✓ |
| Cli.vul | *Clinopodium vulgare* |  | ✓ |  | ✓ |
| Col.nap | *Colchicum napolitanum* |  |  |  | ✓ |
| Col.myc | *Coleostephus myconis* | ✓ |  |  |  |
| Con.maj | *Convallaria majalis* |  |  | ✓ | ✓ |
| Con.arv | *Convolvulus arvensis* | ✓ |  |  |  |
| Con.spe | *Convolvulus sp.* | ✓ |  |  |  |
| Cor.mas | *Cornus mas* |  |  | ✓ |  |
| Cor.san | *Cornus sanguinea* |  |  | ✓ |  |
| Cor.cav | *Corydalis cava* |  |  | ✓ |  |
| Cor.ave | *Corylus avellana* |  |  | ✓ | ✓ |
| Cra.ger | *Crataegus germanica* |  |  |  | ✓ |
| Cra.lae | *Crataegus laevigata* | ✓ |  | ✓ | ✓ |
| Cra.mon | *Crataegus monogyna* | ✓ | ✓ | ✓ | ✓ |
| Cre.cap | *Crepis capillaris* | ✓ | ✓ |  |  |
| Cre.leo | *Crepis leontodontoides* | ✓ |  | ✓ | ✓ |
| Cre.pal | *Crepis paludosa* |  |  |  | ✓ |
| Cre.tec | *Crepis tectorum* | ✓ |  |  |  |
| Cro.etr | *Crocus etruscus* |  |  | ✓ | ✓ |
| Cro.ver | *Crocus vernus* |  |  | ✓ | ✓ |
| Cru.gla | *Cruciata glabra* | ✓ | ✓ | ✓ | ✓ |
| Cyc.hed | *Cyclamen hederifolium* | ✓ |  |  |  |
| Cyc.rep | *Cyclamen repandum* |  |  | ✓ | ✓ |
| Cyn.dac | *Cynodon dactylon* | ✓ | ✓ |  |  |
| Cyt.sco | *Cytisus scoparius* | ✓ | ✓ | ✓ | ✓ |
| Cyt.spe | *Cytisus sp.* | ✓ |  |  |  |
| Dac.glo | *Dactylis glomerata* | ✓ | ✓ | ✓ | ✓ |
| Dan.dec | *Danthonia decumbens* |  |  | ✓ |  |
| Dap.lau | *Daphne laureola* |  |  | ✓ | ✓ |
| Des.ces | *Deschampsia cespitosa* | ✓ | ✓ | ✓ | ✓ |
| Dig.gra | *Digitalis grandiflora* |  | ✓ |  |  |
| Dig.lut.aus | *Digitalis lutea subsp. australis* | ✓ |  | ✓ | ✓ |
| Dig.pur | *Digitalis purpurea* | ✓ |  |  |  |
| Dig.san | *Digitaria sanguinalis* |  | ✓ |  |  |
| Dio.com | *Dioscorea communis* |  |  | ✓ | ✓ |
| Ditt.vis | *Dittrichia viscosa* |  | ✓ |  |  |
| Dor.hir | *Dorycnium hirsutum* |  |  |  | ✓ |
| Dry.car | *Dryopteris carthusiana* |  |  |  | ✓ |
| Dry.dil | *Dryopteris dilatata* |  |  | ✓ |  |
| Dry.exp | *Dryopteris expansa* |  |  | ✓ |  |
| Dry.fil | *Dryopteris filix-mas* |  |  |  | ✓ |
| Emp.nig | *Empetrum nigrum* | ✓ | ✓ |  |  |
| Epi.ang | *Epilobium angustifolium* | ✓ | ✓ | ✓ | ✓ |
| Epi.spe | *Epilobium ciliatum* | ✓ |  |  |  |
| Epi.hir | *Epilobium hirsutum* | ✓ |  |  |  |
| Epi.lan | *Epilobium lanceolatum* | ✓ | ✓ |  |  |
| Epi.mon | *Epilobium montanum* | ✓ | ✓ | ✓ |  |
| Epi.tet | *Epilobium tetragonum* | ✓ | ✓ |  |  |
| Epi.hel | *Epipactis helleborine* |  |  | ✓ | ✓ |
| Equ.pal | *Equisetum palustre* |  |  |  | ✓ |
| Equ.syl | *Equisetum sylvaticum* |  |  | ✓ |  |
| Era.mul | *Eragrostis multicaulis* | ✓ | ✓ |  |  |
| Era.spe | *Eragrostis sp.* |  | ✓ |  |  |
| Eri.arb | *Erica arborea* | ✓ | ✓ | ✓ | ✓ |
| Eri.sco | *Erica scoparia* | ✓ | ✓ |  |  |
| Eri.tet | *Erica tetralix* |  | ✓ |  |  |
| Eri.ann | *Erigeron annuus* | ✓ |  |  |  |
| Eri.can | *Erigeron canadensis* | ✓ | ✓ |  |  |
| Euo.eur | *Euonymus europaeus* |  |  | ✓ |  |
| Eup.can | *Eupatorium cannabinum* | ✓ | ✓ | ✓ |  |
| Eup.amy | *Euphorbia amygdaloides* |  | ✓ | ✓ | ✓ |
| Eup.cyp | *Euphorbia cyparissias* |  |  | ✓ |  |
| Eup.dul | *Euphorbia dulcis* |  |  | ✓ |  |
| Fag.syl | *Fagus sylvatica* |  |  | ✓ | ✓ |
| Fes.het | *Festuca heterophylla* | ✓ |  | ✓ | ✓ |
| Fes.ovi | *Festuca ovina* | ✓ | ✓ | ✓ | ✓ |
| Fes.pal | *Festuca pallens* |  | ✓ |  |  |
| Fes.rub | *Festuca rubra* | ✓ | ✓ |  |  |
| Fic.ver | *Ficaria verna* | ✓ | ✓ | ✓ | ✓ |
| Fil.ulm | *Filipendula ulmaria* |  |  | ✓ | ✓ |
| Fra.ves | *Fragaria vesca* | ✓ | ✓ | ✓ | ✓ |
| Fra.exl | *Fraxinus excelsior* |  |  | ✓ | ✓ |
| Fra.orn | *Fraxinus ornus* |  |  | ✓ | ✓ |
| Fum.off | *Fumaria officinalis* |  | ✓ |  |  |
| Gal.niv | *Galanthus nivalis* |  |  |  | ✓ |
| Gal.pub | *Galeopsis pubescens* |  |  | ✓ | ✓ |
| Gal.tet | *Galeopsis tetrahit* | ✓ |  | ✓ | ✓ |
| Gal.qua | *Galinsoga quadriradiata* | ✓ |  |  |  |
| Gal.alb | *Galium album* | ✓ | ✓ |  |  |
| Gal.apa | *Galium aparine* |  |  | ✓ |  |
| Gal.bor | *Galium boreale* | ✓ |  | ✓ | ✓ |
| Gal.mol | *Galium mollugo* | ✓ |  | ✓ |  |
| Gal.odo | *Galium odoratum* |  | ✓ |  | ✓ |
| Gal.spe | *Galium spec.* |  |  | ✓ |  |
| Gen.ang | *Genista anglica* |  | ✓ |  |  |
| Gen.rad | *Genista radiata* | ✓ |  |  |  |
| Ger.col | *Geranium columbinum* |  | ✓ |  |  |
| Ger.pur | *Geranium purpureum* |  |  | ✓ | ✓ |
| Ger.rob | *Geranium robertianum* | ✓ |  | ✓ |  |
| Ger.rot | *Geranium rotundifolium* |  |  |  | ✓ |
| Ger.syl | *Geranium sylvaticum* |  | ✓ | ✓ | ✓ |
| Geu.riv | *Geum rivale* | ✓ | ✓ | ✓ | ✓ |
| Geu.urb | *Geum urbanum* | ✓ | ✓ | ✓ | ✓ |
| Gle.hed | *Glechoma hederacea* | ✓ |  | ✓ |  |
| Gle.hir | *Glechoma hirsuta* |  |  | ✓ |  |
| Gna.syl | *Gnaphalium sylvaticum* | ✓ | ✓ |  |  |
| Gna.uli | *Gnaphalium uliginosum* | ✓ | ✓ |  |  |
| Gym.dry | *Gymnocarpium dryopteris* |  |  | ✓ | ✓ |
| Hed.hel | *Hedera helix* |  |  | ✓ | ✓ |
| Hel.num | *Helianthemum nummularium* |  |  | ✓ |  |
| Hel.foe | *Helleborus foetidus* |  |  | ✓ |  |
| Hel.vir | *Helleborus viridis* |  |  | ✓ | ✓ |
| Hep.nob | *Hepatica nobilis* |  |  | ✓ | ✓ |
| Her.sph | *Heracleum sphondylium* |  |  | ✓ |  |
| Hie.mur | *Hieracium murorum* | ✓ | ✓ | ✓ | ✓ |
| Hie.rac | *Hieracium racemosum* |  | ✓ |  | ✓ |
| Hie.sab | *Hieracium sabaudum* | ✓ |  | ✓ |  |
| Hie.spe | *Hieracium spec.* |  |  | ✓ |  |
| Hol.lan | *Holcus lanatus* | ✓ |  | ✓ | ✓ |
| Hol.mol | *Holcus mollis* |  |  | ✓ |  |
| Hya.non | *Hyacinthoides non-scripta* |  |  | ✓ | ✓ |
| Hyp.hir | *Hypericum hirsutum* |  | ✓ |  |  |
| Hyp.hum | *Hypericum humifusum* | ✓ |  |  |  |
| Hyp.mac | *Hypericum maculatum* | ✓ | ✓ | ✓ | ✓ |
| Hyp.mon | *Hypericum montanum* | ✓ |  |  |  |
| Hyp.per | *Hypericum perforatum* | ✓ | ✓ | ✓ | ✓ |
| Hyp.rad | *Hypochaeris radicata* | ✓ |  |  |  |
| Ile.aqu | *Ilex aquifolium* |  |  | ✓ | ✓ |
| Imp.par | *Impatiens parviflora* | ✓ |  | ✓ |  |
| Inu.con | *Inula conyzae* | ✓ | ✓ | ✓ |  |
| Iso.set | *Isolepis setacea* | ✓ | ✓ |  |  |
| Jun.buf | *Juncus bufonius* | ✓ | ✓ |  |  |
| Jun.con | *Juncus conglomeratus* | ✓ | ✓ | ✓ |  |
| Jun.eff | *Juncus effusus* | ✓ | ✓ | ✓ |  |
| Jun.inf | *Juncus inflexus* | ✓ | ✓ | ✓ |  |
| Jun.spe | *Juncus sp.* | ✓ | ✓ |  |  |
| Jun.ten | *Juncus tenuis* | ✓ |  |  |  |
| Jun.com | *Juniperus communis* |  |  |  | ✓ |
| Kna.arv | *Knautia arvensis* |  |  | ✓ |  |
| Lac.mur | *Lactuca muralis* | ✓ | ✓ |  | ✓ |
| Lam.gal | *Lamium galeobdolon* | ✓ |  | ✓ | ✓ |
| Lap.com | *Lapsana communis* | ✓ |  | ✓ |  |
| Lat.lin | *Lathyrus linifolius* |  |  | ✓ | ✓ |
| Lat.nig | *Lathyrus niger* |  |  | ✓ |  |
| Lat.pra | *Lathyrus pratensis* | ✓ |  | ✓ |  |
| Lat.ven | *Lathyrus venetus* |  |  |  | ✓ |
| Lat.ver | *Lathyrus vernus* |  |  |  | ✓ |
| Leo.his | *Leontodon hispidus* |  | ✓ |  |  |
| Leo.com | *Leopoldia comosa* |  |  | ✓ |  |
| Leu.pal | *Leucanthemum pallens* | ✓ |  |  |  |
| Leu.vul | *Leucanthemum vulgare* | ✓ |  | ✓ |  |
| Lig.vul | *Ligustrum vulgare* |  |  | ✓ | ✓ |
| Lil.bul | *Lilium bulbiferum* |  |  |  | ✓ |
| Lol.per | *Lolium perenne* | ✓ | ✓ | ✓ |  |
| Lon.etr | *Lonicera etrusca* |  |  | ✓ | ✓ |
| Lon.per | *Lonicera periclymenum* |  |  | ✓ | ✓ |
| Lon.xyl | *Lonicera xylosteum* |  |  | ✓ | ✓ |
| Lot.cor | *Lotus corniculatus* | ✓ | ✓ | ✓ | ✓ |
| Luz.cam | *Luzula campestris* | ✓ | ✓ | ✓ | ✓ |
| Luz.for | *Luzula forsteri* | ✓ | ✓ | ✓ | ✓ |
| Luz.mul | *Luzula multiflora* | ✓ | ✓ | ✓ | ✓ |
| Luz.pil | *Luzula pilosa* | ✓ | ✓ | ✓ |  |
| Luz.syl | *Luzula sylvatica* | ✓ |  |  |  |
| Lys.vul | *Lysimachia vulgaris* |  | ✓ | ✓ | ✓ |
| Mai.bif | *Maianthemum bifolium* |  |  | ✓ | ✓ |
| Mal.spe | *Malus spec.* |  |  | ✓ |  |
| Mal.syl | *Malus sylvestris* |  |  | ✓ | ✓ |
| Mat.cha | *Matricaria chamomilla* | ✓ |  |  |  |
| Med.orb | *Medicago orbicularis* | ✓ |  |  |  |
| Med.spe | *Medicago sp.* | ✓ | ✓ |  |  |
| Mel.pra | *Melampyrum pratense* |  |  | ✓ | ✓ |
| Mel.nut | *Melica nutans* | ✓ | ✓ | ✓ | ✓ |
| Mel.uni | *Melica uniflora* | ✓ |  | ✓ | ✓ |
| Mel.mel | *Melittis melissophyllum* |  |  | ✓ |  |
| Men.arv | *Mentha arvensis* | ✓ |  |  |  |
| Men.pul | *Mentha pulegium* | ✓ |  |  |  |
| Men.spi | *Mentha spicata* | ✓ |  |  |  |
| Men.sua | *Mentha suaveolens* |  | ✓ |  |  |
| Mer.per | *Mercurialis perennis* |  |  | ✓ | ✓ |
| Mil.eff | *Milium effusum* | ✓ | ✓ | ✓ | ✓ |
| Moe.tri | *Moehringia trinervia* | ✓ | ✓ | ✓ | ✓ |
| Mol.cae | *Molinia caerulea* |  |  |  | ✓ |
| Neo.ova | *Neottia ovata* |  |  | ✓ |  |
| Oen.pim | *Oenanthe pimpinelloides* | ✓ |  | ✓ | ✓ |
| Orn.com | *Ornithopus compressus* | ✓ | ✓ |  |  |
| Oxa.ace | *Oxalis acetosella* | ✓ | ✓ | ✓ | ✓ |
| Pan.cap | *Panicum capillare* | ✓ |  |  |  |
| Pap.rho | *Papaver rhoeas* | ✓ | ✓ |  |  |
| Par.qua | *Paris quadrifolia* |  |  | ✓ | ✓ |
| Pet.hyb | *Petasites hybridus* |  |  | ✓ |  |
| Phe.con | *Phegopteris connectilis* |  |  | ✓ | ✓ |
| Phi.ang | *Phillyrea angustifolia* |  |  | ✓ | ✓ |
| Phi.lat | *Phillyrea latifolia* |  |  | ✓ |  |
| Phe.pra | *Phleum pratense* |  |  | ✓ |  |
| Phy.spi | *Phyteuma spicatum* |  |  | ✓ |  |
| Pic.abi | *Picea abies* |  |  | ✓ | ✓ |
| Pic.hie | *Picris hieracioides* | ✓ | ✓ |  |  |
| Pis.his | *Pistorinia hispanica* |  | ✓ |  |  |
| Pla.maj | *Plantago major* | ✓ | ✓ |  |  |
| Pla.chl | *Platanthera chlorantha* |  |  | ✓ |  |
| Poa.ann | *Poa annua* | ✓ | ✓ |  |  |
| Poa.nem | *Poa nemoralis* | ✓ | ✓ | ✓ | ✓ |
| Poa.pra | *Poa pratensis* | ✓ | ✓ | ✓ |  |
| Poa.tri | *Poa trivialis* | ✓ | ✓ | ✓ | ✓ |
| Poa.syl | *Poa trivialis subsp. sylvicola* | ✓ | ✓ | ✓ | ✓ |
| Poa.spe | *Poaceae spec.* |  |  | ✓ | ✓ |
| Pol.fla | *Polygala flavescens* |  |  | ✓ |  |
| Pol.mul | *Polygonatum multiflorum* |  |  | ✓ |  |
| Pol.avi | *Polygonum aviculare* | ✓ |  |  |  |
| Pol.int | *Polypodium interjectum* |  |  |  | ✓ |
| Pop.spe | *Populus spec.* |  |  | ✓ |  |
| Pop.tre | *Populus tremula* |  |  | ✓ | ✓ |
| Por.ole | *Portulaca oleracea* | ✓ | ✓ |  |  |
| Pot.ere | *Potentilla erecta* |  |  | ✓ | ✓ |
| Pot.mic | *Potentilla micrantha* | ✓ | ✓ | ✓ |  |
| Pri.aca | *Primula acaulis* | ✓ | ✓ | ✓ | ✓ |
| Pru.vul | *Prunella vulgaris* | ✓ | ✓ | ✓ |  |
| Pru.avi | *Prunus avium* |  |  | ✓ | ✓ |
| Pru.pad | *Prunus padus* |  | ✓ | ✓ | ✓ |
| Pru.ser | *Prunus serotina* |  |  | ✓ | ✓ |
| Pru.spi | *Prunus spinosa* |  |  | ✓ | ✓ |
| Pte.aqu | *Pteridium aquilinum* |  |  | ✓ | ✓ |
| Pul.dys | *Pulicaria dysenterica* | ✓ |  |  |  |
| Pul.odo | *Pulicaria odora* |  |  | ✓ |  |
| Pul.off | *Pulmonaria officinalis* |  |  | ✓ |  |
| Pyr.min | *Pyrola minor* |  |  | ✓ | ✓ |
| Pyr.com | *Pyrus communis subsp. pyraster* |  |  |  | ✓ |
| Que.cer | *Quercus cerris* |  |  | ✓ | ✓ |
| Que.ile | *Quercus ilex* |  |  | ✓ |  |
| Que.pet | *Quercus petraea* |  |  | ✓ | ✓ |
| Que.rob | *Quercus robur* |  |  | ✓ | ✓ |
| Que.rub | *Quercus rubra* |  |  | ✓ | ✓ |
| Ran.acr | *Ranunculus acris* | ✓ | ✓ | ✓ | ✓ |
| Ran.aur | *Ranunculus auricomus* |  |  | ✓ | ✓ |
| Ran.bul | *Ranunculus bulbosus* | ✓ |  | ✓ |  |
| Ran.lan | *Ranunculus lanuginosus* |  | ✓ |  |  |
| Ran.rep | *Ranunculus repens* | ✓ | ✓ | ✓ | ✓ |
| Ran.sce | *Ranunculus sceleratus* |  | ✓ |  |  |
| Ran.vel | *Ranunculus velutinus* | ✓ |  | ✓ | ✓ |
| Rob.pse | *Robinia pseudoacacia* | ✓ |  |  |  |
| Ros.can | *Rosa canina* |  |  | ✓ | ✓ |
| Ros.sem | *Rosa sempervirens* |  |  | ✓ | ✓ |
| Ros.spe | *Rosa spec.* |  |  |  | ✓ |
| Rub.per | *Rubia peregrina* |  |  | ✓ | ✓ |
| Rub.can | *Rubus canescens* |  |  |  | ✓ |
| Rub.fru | *Rubus fruticosus* | ✓ | ✓ | ✓ | ✓ |
| Rub.hir | *Rubus hirtus* |  | ✓ | ✓ | ✓ |
| Rub.ida | *Rubus idaeus* | ✓ | ✓ | ✓ | ✓ |
| Rub.sax | *Rubus saxatilis* | ✓ | ✓ | ✓ | ✓ |
| Rub.spe | *Rubus spec.* | ✓ | ✓ | ✓ |  |
| Rub.ulm | *Rubus ulmifolius* | ✓ |  | ✓ | ✓ |
| Rum.ace | *Rumex acetosa* |  | ✓ | ✓ |  |
| Rum.obt | *Rumex obtusifolius* | ✓ | ✓ |  |  |
| Rum.san | *Rumex sanguineus* | ✓ |  | ✓ |  |
| Rus.acu | *Ruscus aculeatus* |  |  | ✓ | ✓ |
| Sal.cap | *Salix caprea* |  |  |  | ✓ |
| Sal.spe | *Salix sp.* | ✓ |  |  |  |
| Sam.nig | *Sambucus nigra* | ✓ | ✓ | ✓ |  |
| Sam.spe | *Sambucus spec.* |  |  |  | ✓ |
| San.eur | *Sanicula europaea* |  |  |  | ✓ |
| Sap.off | *Saponaria officinalis* |  | ✓ |  |  |
| Sau.alp | *Saussurea alpina* |  |  |  | ✓ |
| Sch.gig | *Schedonorus giganteus* |  |  |  | ✓ |
| Sci.bif | *Scilla bifolia* |  |  | ✓ | ✓ |
| Sco.mur | *Scorpiurus muricatus* | ✓ |  |  |  |
| Sco.nod | *Scrophularia nodosa* | ✓ | ✓ | ✓ |  |
| Sen.ova | *Senecio ovatus* |  |  | ✓ |  |
| Sen.vis | *Senecio viscosus* |  |  | ✓ |  |
| Sen.vul | *Senecio vulgaris* | ✓ |  |  |  |
| She.arv | *Sherardia arvensis* |  |  | ✓ |  |
| Sil.dio | *Silene dioica* |  |  | ✓ |  |
| Sil.flo | *Silene flos-cuculi* | ✓ |  |  |  |
| Sil.ita | *Silene italica* | ✓ | ✓ | ✓ | ✓ |
| Sol.nig | *Solanum nigrum* | ✓ | ✓ |  |  |
| Sol.gig | *Solidago gigantea* | ✓ | ✓ |  |  |
| Sol.vir | *Solidago virgaurea* |  | ✓ | ✓ | ✓ |
| Son.arv | *Sonchus arvensis* |  |  | ✓ |  |
| Son.asp | *Sonchus asper* | ✓ |  |  |  |
| Son.ole | *Sonchus oleraceus* | ✓ | ✓ |  |  |
| Sor.auc | *Sorbus aucuparia* |  |  | ✓ | ✓ |
| Sor.dom | *Sorbus domestica* |  |  | ✓ | ✓ |
| Sor.tor | *Sorbus torminalis* |  |  | ✓ | ✓ |
| Sta.off | *Stachys officinalis* |  |  | ✓ | ✓ |
| Sta.syl | *Stachys sylvatica* | ✓ | ✓ | ✓ | ✓ |
| Ste.gra | *Stellaria graminea* | ✓ |  |  |  |
| Ste.hol | *Stellaria holostea* | ✓ |  | ✓ | ✓ |
| Ste.med | *Stellaria media* | ✓ | ✓ | ✓ | ✓ |
| Ste.nem | *Stellaria nemorum* | ✓ |  | ✓ | ✓ |
| Sym.bul | *Symphytum bulbosum* |  |  |  | ✓ |
| Tar.off | *Taraxacum officinale* | ✓ |  | ✓ | ✓ |
| Teu.cha | *Teucrium chamaedrys* |  |  | ✓ | ✓ |
| Teu.sco | *Teucrium scorodonia* | ✓ | ✓ | ✓ |  |
| Til.cor | *Tilia cordata* |  |  | ✓ | ✓ |
| Tri.eur | *Trientalis europaea* |  |  | ✓ | ✓ |
| Tri.arv | *Trifolium arvense* | ✓ | ✓ |  |  |
| Tri.aur | *Trifolium aureum* | ✓ | ✓ |  |  |
| Tri.cam | *Trifolium campestre* | ✓ | ✓ |  |  |
| Tri.inc | *Trifolium incarnatum* | ✓ | ✓ |  |  |
| Tri.och | *Trifolium ochroleucon* | ✓ | ✓ |  |  |
| Tri.pra | *Trifolium pratense* | ✓ | ✓ | ✓ |  |
| Tri.rep | *Trifolium repens* | ✓ | ✓ | ✓ |  |
| Tri.spe | *Trifolium sp.* | ✓ |  |  |  |
| Tri.sti | *Trifolium striatum* | ✓ | ✓ |  |  |
| Typ.ang | *Typha angustifolia* | ✓ | ✓ |  |  |
| Ulm.gla | *Ulmus glabra* |  |  | ✓ | ✓ |
| Ulm.min | *Ulmus minor* |  |  | ✓ | ✓ |
| Umb.rup | *Umbilicus rupestris* | ✓ | ✓ |  |  |
| Uro.dal | *Urospermum dalechampii* |  |  | ✓ |  |
| Urt.dio | *Urtica dioica* | ✓ | ✓ | ✓ | ✓ |
| Vac.myr | *Vaccinium myrtillus* | ✓ |  | ✓ | ✓ |
| Vac.vit | *Vaccinium vitis-idaea* |  |  | ✓ | ✓ |
| Val.exc | *Valeriana excelsa subsp. sambucifolia* |  |  | ✓ |  |
| Ver.nig | *Verbascum nigrum* | ✓ |  |  |  |
| Ver.tap | *Verbascum thapsus* | ✓ |  |  |  |
| Ver.tap | *Verbascum thapsus* |  | ✓ |  |  |
| Verb.off | *Verbena officinalis* |  | ✓ |  |  |
| Ver.arv | *Veronica arvensis* | ✓ | ✓ |  |  |
| Ver.bec | *Veronica beccabunga* |  | ✓ |  |  |
| Ver.cha | *Veronica chamaedrys* | ✓ | ✓ | ✓ | ✓ |
| Ver.mon | *Veronica montana* | ✓ | ✓ | ✓ |  |
| Ver.off | *Veronica officinalis* | ✓ | ✓ | ✓ | ✓ |
| Ver.per | *Veronica persica* | ✓ |  | ✓ |  |
| Ver.ser | *Veronica serpyllifolia* | ✓ |  | ✓ |  |
| Vir.lan | *Viburnum lantana* |  |  | ✓ |  |
| Vir.opu | *Viburnum opulus* |  |  |  | ✓ |
| Vic.bit | *Vicia bithynica* |  |  |  | ✓ |
| Vic.cra | *Vicia cracca* |  |  | ✓ |  |
| Vic.hir | *Vicia hirsuta* |  |  | ✓ |  |
| Vic.och | *Vicia ochroleuca* |  |  | ✓ | ✓ |
| Vic.sep | *Vicia sepium* |  | ✓ | ✓ | ✓ |
| Vic.spe | *Vicia spec.* |  |  | ✓ | ✓ |
| Vic.syl | *Vicia sylvatica* |  |  |  | ✓ |
| Vic.tet | *Vicia tetrasperma* |  | ✓ | ✓ |  |
| Vio.alb | *Viola alba* | ✓ |  | ✓ | ✓ |
| Vio.bif | *Viola biflora* |  | ✓ | ✓ | ✓ |
| Vio.rei | *Viola reichenbachiana* |  | ✓ | ✓ | ✓ |
| Vio.riv | *Viola riviniana* | ✓ | ✓ | ✓ | ✓ |
| Vio.spe | *Viola spec.* |  |  | ✓ | ✓ |


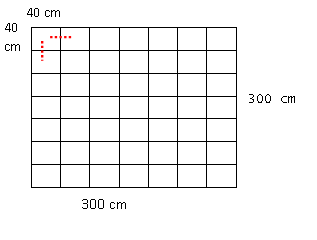

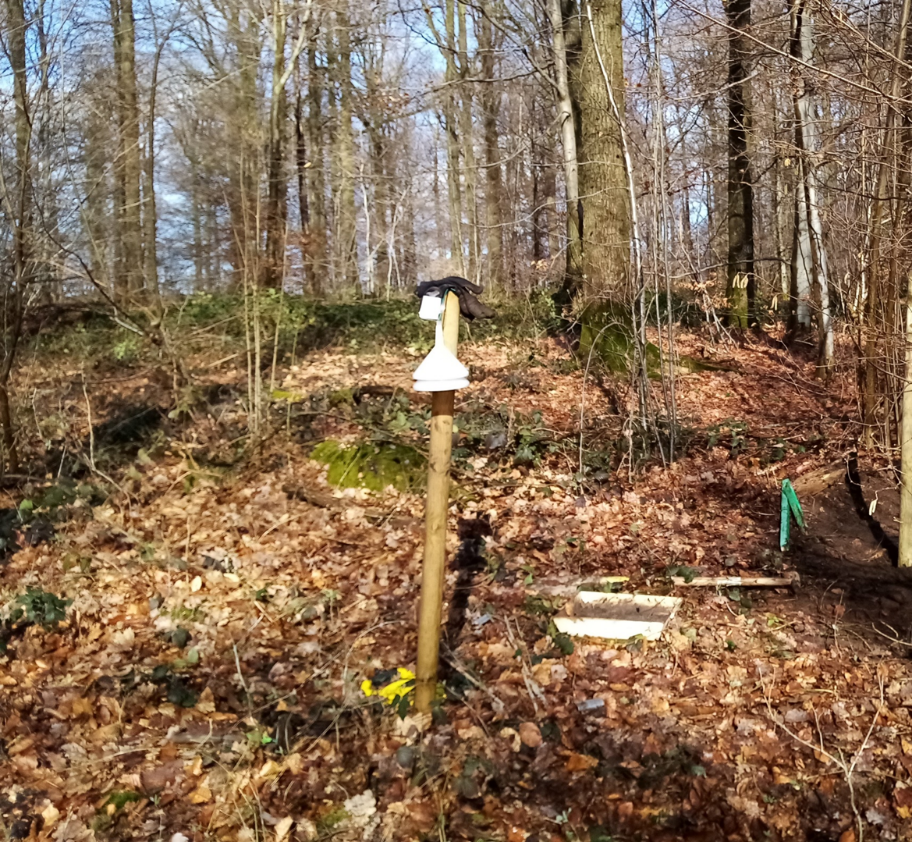


**Figure A2.** The centre of the experimental plot (3 m × 3 m) is represented by the pole with the microclimatic sensors (air=under the white shield, soil=underground). Alongside, the ideal grid followed to systematically collect the soil samples.

**Table A3:** Litter quality scores for tree and shrub species found in the herb-layer (Maes et al., 2019; Vanneste et al., 2020; Verheyen et al., 2012). Values close to one indicate a very low decomposition rate while close to five values have a very high decomposition rate. Scores for species that were not included in any of these sources (e.g. *Juniperus communis, Phillyrea latifolia, Pyrus communis subsp. pyraster*), were derived from literature on the litter quality or decomposition rates of those species.

| **Species** | **Litter quality** |
| --- | --- |
| *Acer campestre* | 4 |
| *Acer monspessulanum* | 3 |
| *Acer platanoides* | 3 |
| *Acer pseudoplatanus* | 3 |
| *Alnus incana* | 3 |
| *Betula pubescens* | 2 |
| *Carpinus betulus* | 3 |
| *Castanea sativa* | 2 |
| *Cornus mas* | 5 |
| *Cornus sanguinea* | 5 |
| *Corylus avellana* | 3 |
| *Crataegus laevigata* | 3 |
| *Crataegus monogyna* | 3 |
| *Cytisus scoparius* | 3 |
| *Erica arborea* | 3 |
| *Fagus sylvatica* | 1 |
| *Fraxinus excelsior* | 5 |
| *Fraxinus ornus* | 5 |
| *Juniperus communis* | 1 |
| *Ligustrum vulgare* | 3 |
| *Malus sylvestris* | 4 |
| *Ostrya carpinifolia* | 3 |
| *Phillyrea latifolia* | 2 |
| *Picea abies* | 1 |
| *Populus tremula* | 3 |
| *Prunus avium* | 4 |
| *Prunus padus* | 4 |
| *Prunus serotina* | 3 |
| *Prunus spinosa* | 5 |
| *Pyrus communis subsp. pyraster* | 4 |
| *Quercus cerris* | 1.5 |
| *Quercus ilex* | 1 |
| *Quercus petraea* | 1.5 |
| *Quercus pubescens* | 1.5 |
| *Quercus robur* | 1 |
| *Quercus rubra* | 1 |
| *Salix caprea* | 5 |
| *Sambucus nigra* | 5 |
| *Sorbus aucuparia* | 3 |
| *Sorbus domestica* | 3 |
| *Sorbus torminalis* | 3 |
| *Tilia cordata* | 4 |
| *Ulmus glabra* | 5 |
| *Ulmus minor* | 5 |
| *Viburnum opulus* | 4 |

**Table A4.** Mean and standard deviation of the predictor variables for the different regions.

| region | Sand  (%) | Soil pH | C:N | litter quality | PAI | forest cover  (%) | soil temperature  (°C) | MAP  (mm/year) |
| --- | --- | --- | --- | --- | --- | --- | --- | --- |
| Italy | 25.74 ± 16.59 | 5.83 ± 0.82 | 15.25 ± 2.60 | 2.01 ± 0.61 | 3.79 ± 0.84 | 69.37 ± 15.04 | 13.06 ± 1.79 | 758.22 ± 106.10 |
| Switzerland | 54.67 ± 5.16 | 5.99 ± 0.82 | 15.71 ± 3.47 | 1.56 ± 0.75 | 5.48 ± 1.51 | 71.07 ± 11.09 | 10.56 ± 0.84 | 913.33 ± 6.12 |
| France | 67.7 ± 32.03 | 4.45 ± 0.22 | 14.71 ± 2.45 | 1.88 ± 0.94 | 3.14 ± 2.64 | 79.5 ± 9.59 | 11.16 ± 0.25 | 682.17 ± 46.70 |
| Belgium | 23.68 ± 12.13 | 4.43 ± 0.41 | 15.4 ± 2.33 | 2.3 ± 0.74 | 4.51 ± 1.77 | 55.69 ± 10.02 | 10.26 ± 0.90 | 970.67 ± 127.44 |
| Poland | 44.42 ± 7.72 | 5.83 ± 0.74 | 17.58 ± 1.97 | 1.64 ± 0.97 | 4.25 ± 1.11 | 83.32 ± 6.17 | 8.97 ± 0.51 | 778 ± 9.80 |
| Germany | 80.47 ± 2.14 | 4.32 ± 0.82 | 19.44 ± 4.22 | 1.63 ± 0.52 | 4.43 ± 1.50 | 64.39 ± 8.89 | 9.23 ± 1.19 | 789.33 ± 2.07 |
| South Sweden | 33.3 ± 12.36 | 4.29 ± 0.34 | 15.96 ± 1.34 | 1.42 ± 0.58 | 5.33 ± 1.08 | 56.07 ± 19.06 | 8.55 ± 0.45 | 731.67 ± 51.87 |
| Central  Sweden | 49.95 ± 24.49 | 5.43 ± 0.14 | 14.13 ± 1.10 | 2.64 ± 0.58 | 3.59 ± 1.74 | 56.89 ± 10.31 | 8.07 ± 0.47 | 537.33 ± 14.21 |
| Norway | 60.62 ± 24.58 | 5.47 ± 0.41 | 14.98 ± 2.36 | 2.97 ± 0.86 | 3.58 ± 1.30 | 58.7 ± 14.21 | 5.31 ± 0.78 | 819.83 ± 321.21 |

**Table A5.** List of species emerged from the seed bank per region. Forest guilds are according to Heinken et al., (2019) with 1.1: species which can be mainly found in the closed forest; 1.2: species which occur typically along forest edges and in forest openings; 2.1: species which can be found in both forest and open vegetation; 2.2: species can be found partly in forest, mainly in open vegetation; and O: true open habitat species. All species belonging to 1.1 and 1.2 were grouped as forest specialists (light and dark green), those belonging to 2.1, 2.2 as generalists (light and dark yellow). Open habitat species (O) are in blue. The symbol ‘- ‘ indicates that a certain species was not observed in the study plots of a certain region; ‘nk’ indicates that the habitat preference is not known for that region while ‘\’ that the taxon does not occur in that region. Regions are: IT– Italy; SW – Switzerland; FR– France; BE (Me + Hi) – Belgium (medium and highland); BE (Lo) – Belgium (lowland); PO – Poland; GE – Germany; SS – South Sweden; CS – Central Sweden; NO – Norway. Asterisk indicates true habitat species not found in Heinken et al., (2019) classification, these were assigned to the category based on expert knowledge. Individuals only determined to the genus level were excluded from the forest guild analysis. Italian species were assigned to a category based on (Pignatti et al., 2017-2019). Species for Switzerland were assigned following the closest region (France). Nomenclature follows (Euro+Med, 2006).

| **Species** | **IT** | **SW** | **FR** | **GE** | **BELO** | **BE (ME+HI)** | **PO** | **CS** | **SS** | **NO** |
| --- | --- | --- | --- | --- | --- | --- | --- | --- | --- | --- |
| ***Aegonychon purpurocaeruleum*** | *2.1* | *-* | *-* | *-* | *-* | *-* | *-* | *-* | *-* | *-* |
| ***Agrostis canina*** | *-* | *-* | *-* | *-* | *-* | *-* | *-* | *-* | *-* | *-* |
| ***Agrostis capillaris*** | *-* | *-* | *-* | *-* | *-* | *-* | *-* | *-* | *-* | *-* |
| ***Agrostis stolonifera*** | *-* | *-* | *2.2* | *-* | *-* | *-* | *-* | *-* | *2.2* | *-* |
| ***Ajuga pyramidalis*** | *-* | *-* | *-* | *-* | *-* | *-* | *-* | *2.1* | *-* | *-* |
| ***Ajuga reptans*** | *-* | *-* | *-* | *-* | *-* | *2.1* | *-* | *-* | *-* | *-* |
| ***Alliaria petiolata*** | *2.1* | *-* | *-* | *-* | *-* | *-* | *-* | *-* | *-* | *-* |
| ***Alnus incana*** | *2.1* | *-* | *-* | *1.1* | *-* | *2.1* | *-* | *-* | *-* | *-* |
| ***Anagallis arvensis**** | *O* | *-* | *-* | *-* | *-* | *-* | *-* | *-* | *-* | *-* |
| ***Anisantha sterilis*** | *-* | *-* | *-* | *-* | *-* | *O* | *O* | *-* | *-* | *-* |
| ***Anthoxanthum odoratum*** | *2.2* | *-* | *-* | *2.1* | *-* | *2.2* | *2.1* | *-* | *2.1* | *2.1* |
| ***Anthriscus sylvestris*** | *-* | *-* | *-* | *-* | *-* | *-* | *-* | *2.2* | *-* | *2.1* |
| ***Aphanes arvensis*** | *2.2* | *-* | *-* | *-* | *-* | *-* | *-* | *-* | *-* | *-* |
| ***Arabidopsis thaliana**** | *-* | *-* | *-* | *-* | *O* | *-* | *-* | *-* | *-* | *-* |
| ***Arenaria serpyllifolia**** | *-* | *-* | *-* | *-* | *-* | *O* | *O* | *-* | *O* | *-* |
| ***Astragalus glycyphyllos*** | *-* | *-* | *-* | *-* | *-* | *-* | *2.2* | *-* | *-* | *-* |
| ***Avena sterilis*** | *-* | *-* | *-* | *-* | *-* | *-* | *-* | *-* | *-* | *O* |
| ***Barbarea vulgaris*** | *2.2* | *-* | *-* | *-* | *-* | *-* | *nk* | *-* | *-* | *-* |
| ***Bellis perennis*** | *-* | *-* | *O* | *O* | *-* | *O* | *-* | *-* | *-* | *-* |
| ***Betula pendula*** | *-* | *-* | *2.1* | *2.1* | *2.1* | *2.1* | *2.1* | *2.1* | *2.1* | *2.1* |
| ***Betula pubescens*** | *-* | *-* | *-* | *2.1* | *2.1* | *2.1* | *-* | *-* | *2.1* | *2.1* |
| ***Brachypodium pinnatum*** | *2.2* | *-* | *-* | *-* | *-* | *-* | *2.2* | *-* | *-* | *-* |
| ***Brachypodium sylvaticum*** | *1.1* | *-* | *-* | *-* | *-* | *-* | *1.1* | *-* | *-* | *1.1* |
| ***Bromus hordeaceus*** | *2.2* | *-* | *-* | *-* | *-* | *-* | *-* | *-* | *-* | *-* |
| ***Bromopsis ramosa*** | *1.2* | *-* | *-* | *-* | *-* | *-* | *-* | *-* | *-* | *-* |
| ***Buddleja davidii*** | *-* | *2.2* | *-* | *-* | *-* | *-* | *-* | *-* | *-* | *-* |
| ***Calamagrostis epigejos*** | *-* | *-* | *2.1* | *-* | *-* | *-* | *-* | *-* | *-* | *-* |
| ***Callitriche stagnalis*** | *-* | *-* | *-* | *-* | *nk* | *-* | *-* | *-* | *-* | *-* |
| ***Calluna vulgaris*** | *-* | *-* | *-* | *-* | *-* | *2.1* | *-* | *-* | *-* | *-* |
| ***Campanula rapunculus*** | *2.1* | *-* | *-* | *-* | *-* | *-* | *-* | *-* | *-* | *-* |
| ***Campanula rotundifolia*** | *-* | *-* | *-* | *-* | *-* | *-* | *-* | *2.1* | *-* | *-* |
| ***Carex digitata*** | *-* | *-* | *1.1* | *-* | *-* | *-* | *-* | *-* | *-* | *-* |
| ***Carex flacca*** | *2.2* | *2.2* | *-* | *-* | *-* | *-* | *2.2* | *-* | *-* | *-* |
| ***Carex hirta*** | *-* | *-* | *-* | *-* | *-* | *-* | *2.2* | *-* | *-* | *-* |
| ***Carex olbiensis*** | *1.1* | *-* | *-* | *-* | *-* | *-* | *-* | *-* | *-* | *-* |
| ***Carex ovalis*** | *-* | *-* | *-* | *-* | *-* | *-* | *-* | *nk* | *-* | *-* |
| ***Carex pallescens*** | *2.1* | *-* | *-* | *2.1* | *-* | *-* | *-* | *2.1* | *2.1* | *2.1* |
| ***Carex pendula*** | *-* | *-* | *1.1* | *-* | *-* | *-* | *-* | *-* | *-* | *-* |
| ***Carex pilosa*** | *-* | *1.1* | *-* | *-* | *-* | *-* | *-* | *-* | *-* | *-* |
| ***Carex pilulifera*** | *-* | *-* | *-* | *-* | *-* | *2.1* | *-* | *-* | *-* | *-* |
| ***Cardamine pratensis*** | *-* | *-* | *-* | *2.1* | *-* | *-* | *-* | *-* | *-* | *-* |
| ***Carex remota*** | *-* | *-* | *1.1* | *1.1* | *-* | *1.1* | *-* | *-* | *1.1* | *-* |
| ***Carex sylvatica*** | *-* | *-* | *1.1* | *-* | *1.1* | *1.1* | *1.1* | *-* | *-* | *-* |
| ***Centaurium erythraea*** | *2.2* | *2.2* | *2.2* | *-* | *-* | *O* | *-* | *-* | *-* | *-* |
| ***Cerastium arvense*** | *2.2* | *-* | *-* | *-* | *-* | *-* | *-* | *-* | *-* | *-* |
| ***Cerastium fontanum*** | *-* | *-* | *-* | *-* | *-* | *-* | *-* | *-* | */* | *2.2* |
| ***Cerastium glomeratum*** | *2.2* | *-* | *-* | *-* | *-* | *-* | *-* | *-* | */* | *-* |
| ***Cerastium semidecandrum*** | *2.2* | *-* | *-* | *-* | *-* | *-* | *-* | *-* | *-* | *-* |
| ***Chaerophyllum temulum*** | *1.2* | *-* | *-* | *-* | *-* | *-* | *-* | *-* | *-* | *-* |
| ***Chenopodium album**** | *O* | *O* | *O* | *O* | *-* | *-* | *-* | *-* | *O* | *-* |
| ***Cichorium intybus**** | *-* | *O* | *-* | *-* | *-* | *-* | *-* | *-* | *-* | *-* |
| ***Cirsium arvense*** | *2.2* | *-* | *2.2* | *2.2* | *-* | *-* | *2.2* | *-* | *2.2* | *O* |
| ***Circaea lutetiana*** | *-* | *-* | *-* | *1.1* | *-* | *-* | *-* | *-* | *-* | *-* |
| ***Cistus salvifolius*** | *2.2* | *-* | *-* | *-* | *-* | *-* | *-* | *-* | *-* | *-* |
| ***Clematis vitalba*** | *-* | *-* | *-* | *-* | *-* | *-* | *-* | *-* | *-* | *2.2* |
| ***Clinopodium vulgare*** | *-* | *-* | *-* | *-* | *-* | *-* | *2.1* | *-* | *-* | *-* |
| ***Coleostephus myconis*** | *2.2* | *-* | *-* | *-* | *-* | *-* | *-* | *-* | *-* | *-* |
| ***Convolvulus arvensis*** | *2.2* | *-* | *-* | *-* | *-* | *-* | *-* | *-* | *-* | *-* |
| ***Crataegus laevigata*** | *2.1* | *-* | *-* | *-* | *-* | *-* | *-* | *-* | *-* | *-* |
| ***Crataegus monogyna*** | *2.1* | *-* | *-* | *-* | *2.1* | *-* | *-* | *-* | *-* | *-* |
| ***Cardamine hirsuta*** | *2.2* | *-* | *-* | *nk* | *-* | *nk* | *-* | *-* | *-* | *-* |
| ***Crepis capillaris*** | *-* | *-* | *-* | *-* | *-* | *nk* | *-* | *-* | *nk* | *-* |
| ***Crepis leontodontoides*** | *1.2* | *-* | *-* | *-* | *-* | *-* | *-* | *-* | *-* | *-* |
| ***Crepis tectorum**** | *-* | *-* | *-* | *-* | *-* | *-* | *-* | *-* | *O* | *-* |
| ***Cruciata glabra*** | *2.1* | *-* | *-* | *-* | *-* | *-* | *-* | *-* | *-* | *-* |
| ***Cyclamen hederifolium*** | *1.1* | *-* | *-* | *-* | *-* | *-* | *-* | *-* | *-* | *-* |
| ***Cynodon dactylon**** | *O* | *O* | *-* | *-* | *-* | *-* | *-* | *-* | *-* | *-* |
| ***Cytisus scoparius*** | *2.2* | *-* | *-* | *-* | *-* | *2.2* | *-* | *-* | *-* | *-* |
| ***Dactylis glomerata*** | *2.2* | *2.2* | *2.2* | *2.2* | *-* | *-* | *2.2* | *2.1* | *2.1* | *2.2* |
| ***Deschampsia cespitosa*** | *-* | *-* | *-* | *-* | *-* | *2.1* | *2.1* | *2.1* | *2.1* | *2.2* |
| ***Digitalis grandiflora*** | *-* | *-* | *-* | *-* | *-* | *-* | *1.2* | *-* | *-* | *-* |
| ***Digitalis lutea subsp. australis*** | *1.1* | *-* | *-* | *-* | *-* | *-* | *-* | *-* | *-* | *-* |
| ***Digitalis purpurea*** | *-* | *-* | *-* | *-* | *-* | *1.2* | *-* | *-* | *-* | *-* |
| ***Digitaria sanguinalis**** | *-* | *O* | *-* | *-* | *-* | *-* | *-* | *-* | *-* | *-* |
| ***Dittrichia viscosa*** | *O* | *-* | *-* | *-* | *-* | *-* | *-* | *-* | *-* | *-* |
| ***Empetrum nigrum*** | *-* | *-* | *-* | *-* | *-* | *-* | *-* | *-* | *-* | *2.1* |
| ***Epilobium angustifolium*** | *-* | *-* | *1.2* | *1.2* | *-* | *1.2* | *-* | *-* | *-* | *2.1* |
| ***Epilobium hirsutum*** | *-* | *-* | *-* | *O* | *2.2* | *-* | *-* | *-* | *-* | *-* |
| ***Epilobium lanceolatum*** | *2.1* | *2.2* | *-* | *-* | *O* | *O* | *-* | *-* | *-* | *-* |
| ***Epilobium montanum*** | *-* | *-* | *2.1* | *-* | *2.1* | *2.1* | *-* | *2.2* | *-* | *2.1* |
| ***Epilobium ciliatum*** | *-* | *-* | *-* | *-* | *-* | *-* | *-* | *-* | *-* | *-* |
| ***Epilobium tetragonum*** | *-* | *-* | *-* | *-* | *-* | *-* | *O* | *-* | *O* | *-* |
| ***Eragrostis multicaulis**** | *-* | *-* | *-* | *O* | *-* | *-* | *-* | *-* | *-* | *-* |
| ***Erigeron annuus*** | *-* | *2.2* | *-* | *-* | *-* | *-* | *-* | *-* | *-* | *-* |
| ***Erica arborea*** | *2.1* | *-* | *-* | *-* | *-* | *-* | *-* | *-* | *-* | *-* |
| ***Erigeron canadensis*** | *2.2* | *-* | *-* | *-* | *O* | *-* | *2.2* | *-* | *-* | *O* |
| ***Erica scoparia*** | *2.1* | *-* | *-* | *-* | *-* | *-* | *-* | *-* | *-* | *-* |
| ***Erica tetralix*** | *-* | *-* | *-* | *2.2* | *-* | *-* | *-* | *-* | *-* | *-* |
| ***Euphorbia amygdaloides*** | *1.1* | *-* | *-* | *-* | *-* | *1.1* | *-* | *-* | *-* | *-* |
| ***Eupatorium cannabinum*** | *2.2* | *-* | *-* | *-* | *-* | *-* | *2.1* | *-* | *-* | *-* |
| ***Festuca heterophylla*** | *1.1* | *-* | *-* | *-* | *-* | *-* | *-* | *-* | *-* | *-* |
| ***Festuca ovina*** | *-* | *-* | *-* | *-* | *-* | *-* | *2.1* | *-* | *-* | *2.2* |
| ***Festuca pallens*** | *-* | *-* | *-* | *-* | *-* | *-* | *O* | *-* | *-* | *-* |
| ***Festuca rubra*** | *-* | *-* | *-* | *-* | *-* | *-* | *-* | *-* | *-* | *2.2* |
| ***Ficaria verna*** | *-* | *-* | *-* | *-* | *-* | *-* | *-* | *-* | *-* | *2.1* |
| ***Fragaria vesca*** | *1.2* | *-* | *-* | *-* | *-* | *1.2* | *-* | *-* | *-* | *-* |
| ***Fumaria officinalis*** | *2.2* | *-* | *-* | *-* | *-* | *-* | *-* | *-* | *-* | *-* |
| ***Galium album*** | *2.1* | *-* | *-* | *-* | *-* | *-* | *-* | *-* | *-* | *-* |
| ***Galium boreale*** | *-* | *-* | *-* | *-* | *-* | *-* | *-* | *2.1* | *-* | *2.2* |
| ***Galium mollugo*** | *-* | *-* | *-* | *-* | *-* | *-* | *-* | *-* | *-* | *2.2* |
| ***Galium odoratum*** | *-* | *-* | *-* | *-* | *-* | *-* | *-* | *-* | *-* | *1.1* |
| ***Galinsoga quadriradiata**** | *-* | *-* | *-* | *O* | *-* | *-* | *-* | *-* | *-* | *-* |
| ***Galeopsis tetrahit*** | *-* | *-* | *-* | *-* | *-* | *-* | *2.1* | *-* | *-* | *-* |
| ***Genista anglica**** | *-* | *-* | *-* | *-* | *-* | *O* | *-* | *-* | *-* | *-* |
| ***Genista radiata*** | *1.1* | *-* | *-* | *-* | *-* | *-* | *-* | *-* | *-* | *-* |
| ***Geranium columbinum*** | *-* | *nk* | *-* | *-* | *-* | *-* | *-* | *-* | *-* | *-* |
| ***Geranium robertianum*** | *-* | *-* | *-* | *-* | *-* | *-* | *-* | *-* | *2.1* | *-* |
| ***Geranium sylvaticum*** | *-* | *-* | *-* | *-* | *-* | *-* | *-* | *-* | *-* | *2.1* |
| ***Geum rivale*** | *-* | *-* | *-* | *-* | *-* | *-* | *-* | *-* | *-* | *2.1* |
| ***Geum urbanum*** | *-* | *-* | *-* | *-* | *-* | *-* | *-* | *-* | *-* | *2.1* |
| ***Glechoma hederacea*** | *-* | *-* | *-* | *-* | *-* | *2.1* | *-* | *-* | *-* | *-* |
| ***Gnaphalium sylvaticum*** | *2.1* | *-* | *-* | *2.1* | *-* | *-* | *-* | *-* | *2.1* | *2.1* |
| ***Gnaphalium uliginosum*** | *-* | *-* | *-* | *-* | *2.2* | *-* | *O* | *-* | *-* | *-* |
| ***Hieracium murorum*** | *2.1* | *-* | *-* | *-* | *-* | *-* | *-* | *-* | *-* | *-* |
| ***Hieracium racemosus*** | *2.1* | *-* | *-* | *-* | *-* | *-* | *-* | *-* | *-* | *-* |
| ***Hieracium sabaudum*** | *-* | *-* | *-* | *-* | *-* | *-* | *2.1* | *-* | *-* | *-* |
| ***Holcus lanatus*** | *-* | *-* | *-* | *-* | *-* | *2.2* | *-* | *-* | *-* | *-* |
| ***Hypericum hirsutum*** | *-* | *-* | *-* | *-* | *-* | *1.2* | *-* | *-* | *-* | *-* |
| ***Hypericum humifusum*** | *-* | *-* | *-* | *-* | *-* | *-* | *-* | *-* | *O* | *-* |
| ***Hypericum maculatum*** | *-* | *-* | *-* | *-* | *-* | *-* | *-* | *-* | *-* | *2.1* |
| ***Hypericum montanum*** | *-* | *-* | *-* | *-* | *-* | *1.2* | *-* | *-* | *2.1* | *-* |
| ***Hypericum perforatum*** | *2.2* | *-* | *2.2* | *-* | *O* | *O* | *2.2* | *-* | *2.2* | *2.2* |
| ***Hypochaeris radicata*** | *2.2* | *-* | *2.2* | *-* | *-* | *-* | *-* | *-* | *-* | *-* |
| ***Impatiens parviflora*** | *-* | *-* | *-* | *-* | *-* | *-* | *1.1* | *-* | *-* | *-* |
| ***Inula conyzae*** | *2.2* | *2.2* | *2.2* | *-* | *-* | *2.2* | *-* | *-* | *-* | *-* |
| ***Isolepis setacea*** | *-* | *-* | *2.2* | *-* | *-* | *-* | *-* | *-* | *-* | *-* |
| ***Juncus bufonius*** | *2.2* | *-* | *-* | *2.2* | *-* | *-* | *-* | *-* | *-* | *-* |
| ***Juncus conglomeratus*** | *-* | *-* | *2.2* | *2.2* | *O* | *O* | *-* | *-* | *2.2* | *-* |
| ***Juncus effusus*** | *-* | *-* | *-* | *2.1* | *2.1* | *2.1* | *-* | *2.1* | *2.1* | *-* |
| ***Juncus inflexus*** | *2.1* | *-* | *-* | *-* | *-* | *-* | *-* | *-* | *-* | *-* |
| ***Juncus tenuis*** | *-* | *-* | *-* | *-* | *-* | *2.2* | *-* | *-* | *2.2* | *-* |
| ***Lactuca muralis*** | *2.2* | *-* | *-* | *-* | *O* | *-* | *2.1* | *-* | *-* | *-* |
| ***Lamium galeobdolon*** | *-* | *-* | *-* | *-* | *-* | *-* | *-* | *-* | *1.1* | *-* |
| ***Lapsana communis*** | *-* | *-* | *-* | *-* | *-* | *2.1* | *2.1* | *2.1* | *-* | *2.1* |
| ***Lathyrus pratensis*** | *-* | *-* | *-* | *-* | *-* | *-* | *-* | *-* | *-* | *2.2* |
| ***Leontodon hispidus*** | *-* | *-* | *-* | *-* | *-* | *O* | *-* | *-* | *-* | *-* |
| ***Leucanthemum pallens*** | *2.2* | *-* | *-* | *-* | *-* | *-* | *-* | *-* | *-* | *-* |
| ***Leucanthemum vulgare*** | *-* | *-* | *-* | *-* | *-* | *-* | *-* | *-* | *-* | *2.2* |
| ***Lolium perenne*** | *2.2* | *-* | *-* | *-* | *nk* | *nk* | *nk* | *-* | *nk* | *-* |
| ***Lotus corniculatus*** | *2.2* | *-* | *-* | *-* | *-* | *O* | *-* | *O* | *-* | *2.2* |
| ***Luzula campestris*** | *-* | *-* | *2.2* | *-* | *-* | *-* | *-* | *-* | *-* | *-* |
| ***Luzula forsteri*** | *1.1* | *-* | *-* | *-* | *-* | *-* | *-* | *-* | *-* | *-* |
| ***Luzula multiflora*** | *-* | *-* | *-* | *-* | *-* | *2.1* | *-* | *-* | *-* | *2.1* |
| ***Luzula pilosa*** | *-* | *-* | *1.1* | *-* | *-* | *1.1* | *1.1* | *1.1* | *-* | *-* |
| ***Luzula sylvatica*** | *1.1* | *-* | *-* | *-* | *-* | *-* | *-* | *-* | *-* | *-* |
| ***Lysimachia vulgaris*** | *-* | *-* | *-* | *-* | *-* | *-* | *2.1* | *-* | *-* | *-* |
| ***Matricaria chamomilla**** | *-* | *-* | *-* | *-* | *-* | *-* | *-* | *-* | *O* | *-* |
| ***Medicago orbicularis*** | *2.2* | *-* | *-* | *-* | *-* | *-* | *-* | *-* | *-* | *-* |
| ***Melica nutans*** | *-* | *-* | *-* | *-* | *-* | *-* | *-* | *-* | *1.1* | *1.1* |
| ***Melica uniflora*** | *1.1* | *-* | *-* | *-* | *-* | *-* | *-* | *-* | *-* | *-* |
| ***Mentha arvensis*** | *-* | *-* | *-* | *-* | *-* | *O* | *-* | *-* | *-* | *-* |
| ***Mentha pulegium*** | *2.2* | *-* | *-* | *-* | *-* | *-* | *-* | *-* | *-* | *-* |
| ***Mentha spicata*** | *2.2* | *-* | *-* | *-* | *-* | *-* | *-* | *-* | *-* | *-* |
| ***Mentha suaveolens*** | *2.2* | *-* | *-* | *-* | *-* | *-* | *-* | *-* | *-* | *-* |
| ***Milium effusum*** | *1.1* | *-* | *1.1* | *1.1* | *-* | *-* | *1.1* | *1.1* | *-* | *1.1* |
| ***Moehringia trinervia*** | *1.1* | *-* | *1.1* | *-* | *-* | *1.1* | *1.1* | *-* | *1.1* | *-* |
| ***Oenanthe pimpinelloides*** | *1.1* | *-* | *-* | *-* | *-* | *-* | *-* | *-* | *-* | *-* |
| ***Ornithopus compressus*** | *2.2* | *-* | *-* | *-* | *-* | *-* | *-* | *-* | *-* | *-* |
| ***Oxalis acetosella*** | *1.1* | *-* | *-* | *-* | *-* | *-* | *-* | *-* | *1.1* | *1.1* |
| ***Panicum capillare**** | *-* | *-* | *-* | *-* | *-* | *-* | *-* | *-* | *O* | *-* |
| ***Papaver rhoeas**** | *O* | *-* | *-* | *-* | *-* | *-* | *-* | *-* | *nk* | *-* |
| ***Picris hieracioides*** | *2.2* | *O* | *O* | *-* | *-* | *-* | *-* | *-* | *-* | *-* |
| ***Pistorinia hispanica*** | *-* | *-* | *-* | *-* | *-* | *-* | *-* | *-* | *-* | *nk* |
| ***Plantago major*** | *2.2* | *2.2* | *2.2* | *O* | *2.2* | *2.2* | *O* | *O* | *-* | *-* |
| ***Poa annua**** | *O* | *-* | *O* | *-* | *-* | *-* | *O* | *-* | *O* | *-* |
| ***Poa nemoralis*** | *-* | *-* | *1.1* | *-* | *-* | *1.1* | *-* | *1.1* | *1.1* | *1.1* |
| ***Poa pratensis*** | *2.2* | *-* | *-* | *-* | *O* | *2.2* | *-* | *2.2* | *2.2* | *-* |
| ***Poa trivialis subsp. sylvicola*** | *1.1* | *-* | *-* | *-* | *-* | *-* | *-* | *-* | *-* | *-* |
| ***Poa trivialis*** | *-* | *-* | *2.1* | *-* | *-* | *2.1* | *-* | *-* | *2.1* | *2.2* |
| ***Polygonum aviculare**** | *-* | *-* | *-* | *-* | *-* | *-* | *-* | *-* | *O* | *-* |
| ***Portulaca oleracea*** | *2.2* | *nk* | *-* | *-* | *-* | *-* | *-* | *-* | *-* | *-* |
| ***Potentilla micrantha*** | *1.2* | *-* | *-* | *-* | *-* | *-* | *-* | *-* | *-* | *-* |
| ***Primula acaulis*** | *2.1* | *-* | *-* | *-* | *-* | *-* | *-* | *-* | *-* | *-* |
| ***Prunus padus*** | *-* | *-* | *2.1* | *-* | *-* | *-* | *-* | *-* | *-* | *-* |
| ***Prunella vulgaris*** | *2.2* | *2.2* | *-* | *-* | *-* | *-* | *-* | *-* | *-* | *2.2* |
| ***Pulicaria dysenterica*** | *2.2* | *-* | *-* | *-* | *-* | *-* | *-* | *-* | *-* | *-* |
| ***Ranunculus bulbosus*** | *2.1* | *-* | *-* | *-* | *-* | *-* | *-* | *-* | *-* | *-* |
| ***Ranunculus lanuginosus*** | *2.1* | *-* | *-* | *-* | *-* | *-* | *-* | *-* | *-* | *-* |
| ***Ranunculus repens*** | *-* | *-* | *-* | *2.1* | *2.1* | *2.1* | *2.1* | *-* | *-* | *2.1* |
| ***Ranunculus sceleratus*** | *-* | *-* | *-* | *nk* | *-* | *-* | *-* | *-* | *-* | *-* |
| ***Ranunculus velutinus*** | *2.1* | *-* | *-* | *-* | *-* | *-* | *-* | *-* | *-* | *-* |
| ***Robinia pseudoacacia*** | *2.2* | *-* | *-* | *-* | *-* | *-* | *-* | *-* | *-* | *-* |
| ***Rubus fruticosus*** | *-* | *2.1* | *2.1* | *-* | *2.1* | *2.1* | *2.1* | *-* | *2.1* | *-* |
| ***Rubus hirtus*** | *2.1* | *-* | *-* | *-* | *-* | *-* | *-* | *-* | *-* | *-* |
| ***Rubus idaeus*** | *-* | *1.2* | *-* | *-* | *-* | *1.2* | *1.2* | *-* | *2.1* | *2.1* |
| ***Rubus saxatilis*** | *-* | *-* | *-* | *-* | *-* | *-* | *-* | *2.1* | *-* | *2.1* |
| ***Rubus ulmifolius*** | *2.1* | *-* | *-* | *-* | *-* | *-* | *-* | *-* | *-* | *-* |
| ***Rumex acetosa*** | *-* | *-* | *-* | *2.2* | *-* | *O* | *-* | *-* | *-* | *-* |
| ***Rumex obtusifolius*** | *-* | *2.2* | *-* | *-* | *-* | *-* | *-* | *-* | *2.2* | *-* |
| ***Rumex sanguineus*** | *-* | *-* | *-* | *-* | *-* | *-* | *-* | *-* | *1.1* | *-* |
| ***Sambucus nigra*** | *-* | *-* | *2.1* | *2.1* | *-* | *-* | *2.1* | *-* | *2.1* | *-* |
| ***Saponaria officinalis*** | *-* | *-* | *-* | *-* | *-* | *O* | *-* | *-* | *-* | *-* |
| ***Scorpiurus muricatus*** | *2.2* | *-* | *-* | *-* | *-* | *-* | *-* | *-* | *-* | *-* |
| ***Scrophularia nodosa*** | *1.1* | *1.1* | *1.1* | *-* | *-* | *1.1* | *2.1* | *-* | *-* | *-* |
| ***Senecio vulgaris*** | *2.2* | *-* | *-* | *-* | *-* | *-* | *-* | *-* | *-* | *-* |
| ***Silene flos-cuculi*** | *-* | *-* | *-* | *-* | *-* | *-* | *2.2* | *-* | *-* | *-* |
| ***Silene italica*** | *1.2* | *-* | *-* | *-* | *-* | *-* | *-* | *-* | *-* | *-* |
| ***Solidago gigantea*** | *-* | *-* | *-* | *-* | *-* | *-* | *2.2* | *-* | *-* | *-* |
| ***Solanum nigrum**** | *-* | *O* | *O* | *O* | *-* | *-* | *O* | *-* | *-* | *O* |
| ***Solidago virgaurea*** | *nk* | *-* | *2.1* | *-* | *-* | *-* | *2.1* | *-* | *-* | *-* |
| ***Sonchus asper**** | *O* | *O* | *-* | *-* | *-* | *-* | *-* | *-* | *-* | *-* |
| ***Sonchus oleraceus*** | *2.2* | *-* | *-* | *-* | *-* | *nk* | *-* | *-* | *nk* | *nk* |
| ***Stachys sylvatica*** | *nk* | *-* | *-* | *-* | *1.2* | *1.2* | *-* | *1.1* | *-* | *-* |
| ***Stellaria graminea*** | *nk* | *-* | *-* | *-* | *-* | *-* | *-* | *2.2* | *-* | *2.2* |
| ***Stellaria holostea*** | *nk* | *-* | *-* | *-* | *2.1* | *2.1* | *-* | *-* | *-* | *-* |
| ***Stellaria media*** | *2.2* | *-* | *2.2* | *-* | *2.2* | *-* | *2.1* | *-* | *2.1* | *-* |
| ***Stellaria nemorum*** | *nk* | *-* | *-* | *-* | *-* | *-* | *-* | *-* | *-* | *1.1* |
| ***Ranunculus acris*** | *nk* | *-* | *-* | *-* | *-* | *-* | *-* | *-* | *-* | *2.2* |
| ***Taraxacum officinale*** | *2.2* | *-* | *-* | *-* | *-* | *-* | *-* | *-* | *-* | *nk* |
| ***Teucrium scorodonia*** | *2.1* | *-* | *-* | *-* | *-* | *-* | *-* | *-* | *-* | *-* |
| ***Trifolium arvense*** | *2.2* | *-* | *-* | *-* | *-* | *-* | *-* | *-* | *-* | *-* |
| ***Trifolium aureum*** | *2.1* | *-* | *-* | *-* | *-* | *-* | *-* | *-* | *-* | *-* |
| ***Trifolium campestre*** | *2.2* | *-* | *-* | *-* | *-* | *-* | *-* | *-* | *-* | *-* |
| ***Trifolium incarnatum*** | *2.2* | *-* | *-* | *-* | *-* | *-* | *-* | *-* | *-* | *-* |
| ***Trifolium ochroleucon*** | *1.2* | *-* | *-* | *-* | *-* | *-* | *-* | *-* | *-* | *-* |
| ***Trifolium pratense*** | *2.2* | *-* | *-* | *-* | *-* | *-* | *-* | *-* | *-* | *-* |
| ***Trifolium repens*** | *2.2* | *-* | *-* | *-* | *O* | *-* | *-* | *2.2* | *-* | *2.2* |
| ***Trifolium striatum*** | *2.2* | *-* | *-* | *-* | *-* | *-* | *-* | *-* | *-* | *-* |
| ***Typha angustifolia*** | *-* | *-* | *-* | *-* | *-* | *nk* | *-* | *-* | *nk* | *-* |
| ***Umbilicus rupestris**** | *-* | *-* | *-* | *-* | *-* | *-* | *-* | *-* | *-* | *O* |
| ***Urtica dioica*** | *2.1* | *-* | *2.1* | *2.1* | *2.1* | *2.1* | *-* | *-* | *2.1* | *2.1* |
| ***Vaccinium myrtillus*** | *-* | *-* | *-* | *-* | *-* | *2.1* | *-* | *-* | *-* | *-* |
| ***Veronica arvensis*** | *2.2* | *nk* | *-* | *-* | *-* | *-* | *-* | *-* | *-* | *-* |
| ***Veronica beccabunga*** | *-* | *-* | *-* | *-* | *-* | *-* | *-* | *-* | *-* | *2.2* |
| ***Veronica chamaedrys*** | *2.2* | *-* | *-* | *-* | *-* | *-* | *-* | *2.1* | *2.1* | *2.1* |
| ***Veronica montana*** | *-* | *-* | *-* | *-* | *-* | *-* | *1.1* | *-* | *-* | */* |
| ***Verbascum nigrum*** | *-* | *-* | *-* | *-* | *-* | *-* | *2.2* | *-* | *-* | *-* |
| ***Verbena officinalis*** | *2.2* | *-* | *-* | *-* | *-* | *-* | *-* | *-* | *-* | *-* |
| ***Veronica officinalis*** | *1.2* | *2.1* | *-* | *-* | *-* | *1.2* | *2.1* | *-* | *2.1* | *2.1* |
| ***Veronica persica*** | *2.2* | *-* | *-* | *-* | *-* | *-* | *-* | *-* | *-* | *-* |
| ***Veronica serpyllifolia*** | *-* | *-* | *-* | *-* | *-* | *-* | *-* | *O* | *-* | *-* |
| ***Verbascum thapsus*** | *-* | *2.2* | *-* | *-* | *-* | *O* | *-* | *-* | *-* | *O* |
| ***Vicia sepium*** | *-* | *-* | *-* | *-* | *-* | *-* | *-* | *-* | *-* | *2.2* |
| ***Vicia tetrasperma*** | *2.1* | *-* | *-* | *-* | *-* | *-* | *-* | *-* | *-* | *-* |
| ***Viola alba*** | *1.2* | *-* | *-* | *-* | *-* | *-* | *-* | *-* | *-* | *-* |
| ***Viola biflora*** | *-* | *-* | *-* | *-* | *-* | *-* | *-* | *-* | *-* | *2.1* |
| ***Viola reichenbachiana*** | *1.1* | *-* | *-* | *-* | *-* | *-* | *-* | *-* | *-* | *-* |
| ***Viola riviniana*** | *-* | *-* | *-* | *-* | *-* | *-* | *-* | *-* | *-* | *1.1* |

**Table A6.** Excluded tree and shrub species from the soil seed bank for the community temperature index calculation

| ***Seed-bank*** |
| --- |
| *Alnus incana* |
| *Betula pubescens* |
| *Crataegus laevigata* |
| *Crataegus monogyna* |
| *Cytisus scoparius* |
| *Erica arborea* |
| *Prunus serotina* |
| *Rubus hirtus* |
| *Rubus fruticosus* |
| *Rubus ulmifolius* |
| *Rubus idaeus* |
| *Rubus saxatilis* |
| *Sambucus nigra* |

**Table A7.** Excluded tree and shrub from the herb layer for the community temperature index calculation

| ***Herb -layer*** |  |  |
| --- | --- | --- |
| *Acer campestre* | *Malus sylvestris* | *Rubus fruticosus* |
| *Acer monspessulanum* | *Ostrya carpinifolia* | *Rubus ulmifolius* |
| *Acer pseudoplatanus* | *Phillyrea angustifolia* | *Rubus idaeus* |
| *Acer platanoides* | *Phillyrea latifolia* | *Rubus saxatilis* |
| *Alnus incana* | *Ligustrum vulgare* | *Salix caprea* |
| *Betula pubescens* | *Picea abies* | *Sambucus nigra* |
| *Carpinus betulus* | *Populus tremula* | *Sorbus domestica* |
| *Castanea sativa* | *Prunus avium* | *Sorbus torminalis* |
| *Clematis vitalba* | *Prunus padus* | *Sorbus aucuparia* |
| *Corylus avellana* | *Prunus serotina* | *Tilia cordata* |
| *Cornus mas* | *Prunus spinosa* | *Ulmus minor* |
| *Cornus sanguinea* | *Pyrus communis subsp_pyraster* | *Ulmus glabra* |
| *Crataegus germanica* | *Quercus cerris* | *Fagus sylvatica* |
| *Crataegus laevigata* | *Quercus ilex* | *Viburnum lantana* |
| *Crataegus monogyna* | *Quercus petraea* | *Viburnus opulus* |
| *Cytisus scoparius* | *Quercus robur* |  |
| *Erica arborea* | *Quercus rubra* |  |
| *Euonymus europaeus* | *Rosa canina* |  |
| *Fraxinus ornus* | *Rosa sempervirens* |  |
| *Fraxinus excelsior* | *Rubus canescens* |  |
| *Juniperus communis* | *Rubus hirtus* |  |

**Table A8**. Excluded species from trait analysis.

| **Excluded Species** | **N. of**  **plots** | | **% of plots** |
| --- | --- | --- | --- |
| *Aegonychon purpurocaeruleum* | 1 | 1.1 | |
| *Anisantha sterilis* | 3 | 3.3 | |
| *Bromopsis ramosa* | 1 | 1.1 | |
| *Carex ovalis* | 1 | 1.1 | |
| *Cistus salvifolius* | 2 | 2.2 | |
| *Cynodon dactylon* | 1 | 1.1 | |
| *Poa trivialis subsp. sylvicola* | 4 | 4.4 | |
| *Primula acaulis* | 3 | 3.3 | |

**Table A9.** Mean and standard deviation of the response variables for the different regions.

| region | Seed bank  density | Seed bank  species  richness | Seed bank  richness/  herb layer  richness | Seed bank  shannon | Seed bank  specialist  richness | Seed bank  generalist  richness | Seed bank  proportion  of specialist | Seed bank  CWM  temperature  index | Seed bank  CWM height | Seed bank  CWM SLA | Seed bank  CWM seed mass |
| --- | --- | --- | --- | --- | --- | --- | --- | --- | --- | --- | --- |
| Italy | 69.55 ± 60.37 | 16 ± 5.97 | 0.71 ± 0.26 | 2.2 ± 0.45 | 3.67 ± 2.28 | 11.39 ± 4.38 | 0.22 ± 0.12 | 9.22 ± 0.61 | 1.07 ± 0.97 | 21.67 ± 7.12 | 1.14 ± 1.80 |
| Switzerland | 14.83 ± 13.82 | 6.17 ± 3.76 | 0.63 ± 0.31 | 1.28 ± 0.67 | 1 ± 0.89 | 3 ± 1.90 | 0.05 ± 0.09 | 8.09 ± 1.16 | 0.7 ± 0.30 | 22.28 ± 2.32 | 2.01 ± 1.41 |
| France | 60.17 ± 27.16 | 8.83 ± 2.93 | 0.91 ± 0.25 | 1.41 ± 0.59 | 1.83 ± 1.72 | 5.83 ± 2.04 | 0.23 ± 0.19 | 8.06 ± 0.36 | 0.75 ± 0.15 | 13.91 ± 5.91 | 0.58 ± 0.67 |
| Belgium | 48.06 ± 56.92 | 9.61 ± 2.77 | 1.64 ± 1.22 | 1.3 ± 0.57 | 2 ± 1.71 | 4.56 ± 1.69 | 0.19 ± 0.14 | 7.75 ± 1.11 | 0.79 ± 0.25 | 18.41 ± 7.2 | 0.62 ± 0.72 |
| Poland | 87 ± 60.41 | 14.83 ± 4.17 | 1.84 ± 2.05 | 2.02 ± 0.45 | 2.5 ± 0.55 | 8.5 ± 3.27 | 0.18 ± 0.06 | 7.49 ± 0.81 | 0.95 ± 0.37 | 27.51 ± 3.73 | 0.94 ± 0.38 |
| Germany | 73.83 ± 76.45 | 7.5 ± 5.86 | 1.13 ± 0.78 | 1.07 ± 0.53 | 1.17 ± 1.17 | 4 ±3.03 | 0.15 ± 0.12 | 6.85 ± 1.40 | 0.62 ± 0.23 | 23.34 ± 8.40 | 0.42 ± 0.35 |
| South  Sweden | 126.17 ± 101.52 | 13.33 ± 5.57 | 1.93 ± 0.96 | 1.47 ± 0.62 | 1.67 ± 1.21 | 7.83 ± 2.14 | 0.12 ± 0.04 | 7.16 ± 1.25 | 0.84 ± 0.28 | 19.58 ± 7.23 | 0.44 ± 0.29 |
| Central  Sweden | 22.83 ± 24.00 | 7.17 ± 3.66 | 0.46 ± 0.16 | 1.51 ± 0.41 | 0.83 ± 0.98 | 5 ± 2.28 | 0.1 ± 0.11 | 7.16 ± 0.95 | 0.48 ± 0.14 | 24.1 ± 5.31 | 0.99 ± 0.71 |
| Norway | 67.78 ± 52.53 | 11.22 ± 3.21 | 0.60 ± 0.17 | 1.81 ± 0.39 | 1.61 ± 1.19 | 8 ± 2.83 | 0.13 ± 0.09 | 6.88 ± 0.76 | 0.5 ± 0.34 | 28.37 ± 3.91 | 0.65 ± 0.36 |

**Table A10.**Variables explanation

| **Independent variables** |  |
| --- | --- |
| Latitude | Latitude |
| Elevation | continuous variable: height above sea level (m) |
| Forest type | category with 3 levels, for each structural forest type. T1 = dense, T2 = intermediate, T3 = open |
| Plot | category with 2 levels: edge plot and interior plot |
| % sand | % of sand in pooled soil sample taken at depth of 10-20cm |
| pH | pH-H2O in pooled soil sample taken a depth 0-10cm |
| C:N ratio | carbon/nitrogen ratio in pooled soil sample taken a depth 0-10cm |
| Litter quality | the litter quality was determined based on tree species scores |
| PAI | Plant area index: total area of woody (e.g. branches and stems) and non-woody biomass (i.e. leaves) |
| Soil temperature | (°C) mean annual soil temperature from Lascar temperature loggers |
| MAP | (mm year^-1^) mean precipitation of the period 1979-2013 (from CHELSA version 1.2) |
| Forest cover % | percentage area with a tree cover >20% within a radius of 500 m based on satellite-based global tree cover data |
| **Random effects** |  |
| region | category with 9 levels, for each region of the latitudinal gradient |
| transect | category with 45 levels (region, elevation and forest type) |
| **Dependent variables** |  |
|  |  |
| Seed bank density | seed bank density: number of total emerged seedlings |
| Seed bank species richness | number of plant species emerged from the seed bank |
| Seed bank shannon | Shannon diversity index of the seed bank |
| Seed bank evenness | Pielou's evenness index of the seed bank |
| Seed bank generalist richness | number of generalist species in the seed bank (following Heinken's forest species categorisation) |
| Seed bank specialists richness | number of forest specialists species in the seed bank (following Heinken's forest species categorisation) |
| Seed bank proportion of specialists | percentage of forest species weighted on total species richness in the seed bank |
| Seed bank CWM temperature index | mean of the species temperature preference of the seed bank (°C)source: ClimPlant |
| Seed bank CWM seed mass | mean of the species seed mass from the seed bank (mg) source: D-3, LEDA, BiolFlor, SID, Ecological Flora, Brot 2.0 |
| Seed bank CWM height | mean of the species height from the seed bank (m) source: D-3, LEDA, BiolFlor, SID, Ecological Flora, Brot 2.0 |
| Seed bank CWM sla | mean of the species SLA from the seed bank (mm2/mg) source: LEDA, Ecological Flora, Rothmaler, O, Brot 2.0 |
| Herb layer species richness | number of plant species in the herb layer |
| Herb layer shannon | Shannon diversity index of the herb layer |
| Herb layer evenness | Pielou's evenness index of the herb layer |
| Herb layer generalist richness | number of generalist species in the herb layer (following Heinken's forest species categorisation) |
| Herb layer specialist richnes | number of forest specialists species in the herb layer (following Heinken's forest species categorisation) |
| Herb layer proportion of specialists | percentage of forest species weighted on total species richness in the herb layer |
| Herb layer CWM temperature index | mean of the species temperature preference of the herb layer (°C) source: ClimPlant |
| Herb layer CWM seed mass | mean of the species seed mass from the herb layer (mg) source: D-3, LEDA, BiolFlor, SID, Ecological Flora, Brot 2.0 |
| Herb layer CWM height | mean of the species height from the herb layer (m) source: D-3, LEDA, BiolFlor, SID, Ecological Flora, Brot 2.0 |
| Herb layer CWM sla | mean of the species SLA from the herb layer (mm2/mg) source: LEDA, Ecological Flora, Rothmaler, O, Brot 2.0 |

**References**

Euro+Med, 2006. Euro+Med PlantBase - the information resource for Euro-Mediterranean plant diversity. URL http://ww2.bgbm.org/EuroPlusMed/query.asp

Govaert, S., Meeussen, C., Vanneste, T., Bollmann, K., Brunet, J., Cousins, S.A.O., Diekmann, M., Graae, B.J., Hedwall, P.O., Heinken, T., Iacopetti, G., Lenoir, J., Lindmo, S., Orczewska, A., Perring, M.P., Ponette, Q., Plue, J., Selvi, F., Spicher, F., Tolosano, M., Vermeir, P., Zellweger, F., Verheyen, K., Vangansbeke, P., De Frenne, P., 2020. Edge influence on understorey plant communities depends on forest management. J. Veg. Sci. 31, 281–292. https://doi.org/10.1111/jvs.12844

Heinken, T., Diekmann, M., Liira, J., Orczewska, A., Brunet, J., Chytrý, M., Wulf, . . . M., 2019. European forest plant species list. https://doi.org/https://doi.org/10.6084/m9.figshare.8095217

Karger, D.N., Conrad, O., Böhner, J., Kawohl, T., Kreft, H., Soria-Auza, R.W., Zimmermann, N.E., Linder, H.P., Kessler, M., 2017. Climatologies at high resolution for the earth’s land surface areas. Sci. Data 4, 170122. https://doi.org/10.1038/sdata.2017.122

Maes, S.L., Blondeel, H., Perring, M.P., Depauw, L., Brūmelis, G., Brunet, J., Decocq, G., den Ouden, J., Härdtle, W., Hédl, R., Heinken, T., Heinrichs, S., Jaroszewicz, B., Kirby, K., Kopecký, M., Máliš, F., Wulf, M., Verheyen, K., 2019. Litter quality, land-use history, and nitrogen deposition effects on topsoil conditions across European temperate deciduous forests. For. Ecol. Manage. 433, 405–418. https://doi.org/10.1016/j.foreco.2018.10.056

Meeussen, C., Govaert, S., Vanneste, T., Calders, K., Bollmann, K., Brunet, J., Cousins, S.A.O., Diekmann, M., Graae, B.J., Hedwall, P.-O., Krishna Moorthy, S.M., Iacopetti, G., Lenoir, J., Lindmo, S., Orczewska, A., Ponette, Q., Plue, J., Selvi, F., Spicher, F., Tolosano, M., Verbeeck, H., Verheyen, K., Vangansbeke, P., De Frenne, P., 2020. Structural variation of forest edges across Europe. For. Ecol. Manage. 462, 117929. https://doi.org/10.1016/j.foreco.2020.117929

Pignatti, S., Guarino, R., La Rosa, M., 2017. Flora d’Italia, 2nd Edition. Edagricole-New Business Media.

Vanneste, T., Govaert, S., De Kesel, W., Van Den Berge, S., Vangansbeke, P., Meeussen, C., Brunet, J., Cousins, S.A.O., Decocq, G., Diekmann, M., Graae, B.J., Hedwall, P.O., Heinken, T., Helsen, K., Kapás, R.E., Lenoir, J., Liira, J., Lindmo, S., Litza, K., Naaf, T., Orczewska, A., Plue, J., Wulf, M., Verheyen, K., De Frenne, P., 2020. Plant diversity in hedgerows and road verges across Europe. J. Appl. Ecol. 57, 1244–1257. https://doi.org/10.1111/1365-2664.13620

Verheyen, K., Baeten, L., De Frenne, P., Bernhardt-Römermann, M., Brunet, J., Cornelis, J., Decocq, G., Dierschke, H., Eriksson, O., Hédl, R., Heinken, T., Hermy, M., Hommel, P., Kirby, K., Naaf, T., Peterken, G., Petřík, P., Pfadenhauer, J., Van Calster, H., Walther, G.R., Wulf, M., Verstraeten, G., 2012. Driving factors behind the eutrophication signal in understorey plant communities of deciduous temperate forests. J. Ecol. 100, 352–365. https://doi.org/10.1111/j.1365-2745.2011.01928.x
