# Supplementary material for: Soil seed bank responses to edge effects in temperate European forests
Source: Glob Ecol Biogeogr. 2022 Jul 16;31(9):1877–93. doi: 10.1111/geb.13568 (PMC9546374; doi:10.1111/geb.13568)
Supplement: Supplementary file 2 — Supinfo S2 [file GEB-31-1877-s002.docx]

Appendix B: Supplementary materials- Results


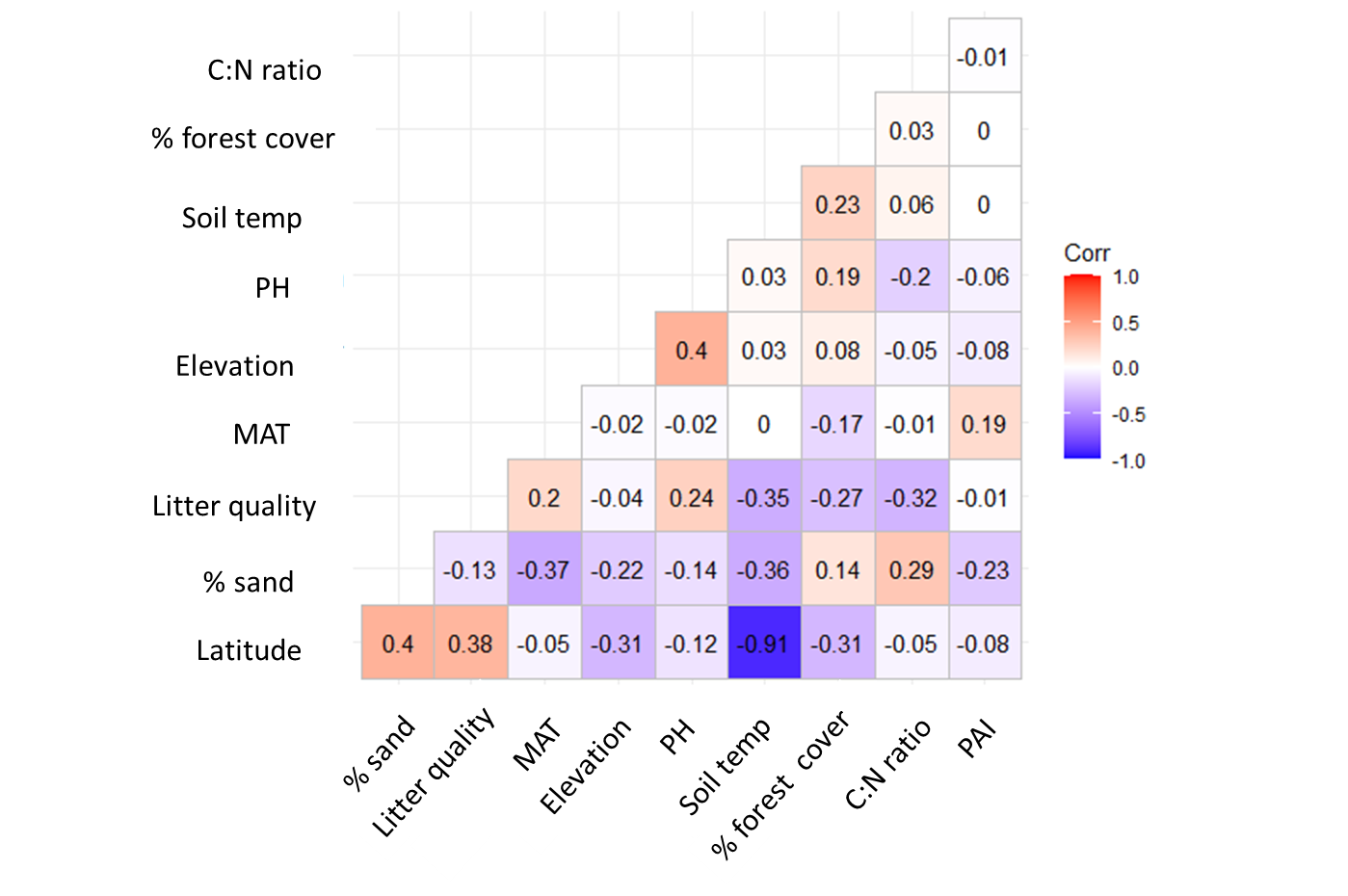


**Figure B1.** Pearson correlations amongst the continuous predictor variables used in the mixed effect models. The value of the correlation is given as a number and indicated by the colour. For an extended description of variables see Table A10.

**Table B1.** Summary of the results of linear mixed models testing the influence of the four design variables (latitude, elevation, forest type and edge vs. interior) on edaphic (C:N, litter quality, % of sand and soil pH), climatic (MAP= mean annual precipitation and microclimate soil temperature) and landscape (PAI=Plant area index and % forest cover=proportion of forest in a radius of 500 m) conditions. Edge plot and dense forest were used as reference categories for edge vs interior and forest type, respectively. Values are parameter estimates after model selection, positive or negative values denote the direction of the effect. The significance of p values is given with *** for p < 0.001; ** for p < 0.01; * for p < 0.05. Model fit was assessed based on marginal R^2^, the proportion of variance explained by fixed effects and conditional R^2^, the proportion of variance explained by both random and fixed effects (Nakagawa and Schielzeth, 2013). For variables explanation see Table A10.

|  | **Edge vs**  **Interior** |  | **Latitude** | **Elevation** | **Intermediate**  **forest**  **type** | **Open**  **forest**  **type** | **R2 marginal** | **R2 conditional** |
| --- | --- | --- | --- | --- | --- | --- | --- | --- |
| C:N |  |  |  |  | -0.394 | 0.832 | 0.03 | 0.27 |
| Litter quality |  |  | 0.406 |  | -0.134 | -0.566* | 0.20 | 0.44 |
| % sand | 0.1258 |  |  |  |  |  | 0.01 | 0.86 |
| soil pH | -0.3069* |  |  |  |  |  | 0.02 | 0.69 |
| MAP |  |  |  | 0.139 |  |  | 0.02 | 0.99 |
| Soil temperature |  |  | -0.989*** | -0.329*** |  |  | 0.88 | 0.94 |
| % forest cover | 2.047** |  | -0.573 |  |  |  | 0.13 | 0.96 |
| PAI | 0.7833*** |  |  |  | 0.222 | -0.389 | 0.21 | 0.52 |

**Table B2.** Summary of the results of generalized linear mixed models testing the influence of the four design variables (latitude, elevation, forest type, edge vs interior) on the abundance and diversity of the herb layer. Edge plot and dense forest were used as reference categories for edge vs interior and forest type, respectively. Values are parameter estimates after model selection, positive or negative values denote the direction of the effect. The significance of p values is given with *** for p < 0.001; ** for p < 0.01; * for p < 0.05. Model fit was assessed based on marginal R^2^, the proportion of variance explained by fixed effects and conditional R^2^, the proportion of variance explained by both random and fixed effects (Nakagawa and Schielzeth, 2013). For variables explanation see Table A10.

|  | **Edge vs**  **interior** | **Latitude** | **Elevation** | **Intermediate**  **forest**  **type** | **Open**  **forest**  **type** |  | **R2 marginal** | **R2 conditional** |
| --- | --- | --- | --- | --- | --- | --- | --- | --- |
| Herb layer species richness | -0.285*** |  |  |  |  |  | 0.07 | 0.75 |
| Herb layer shannon | -0.186 |  |  |  |  |  | 0.02 | 0.52 |
| Herb layer evenness | 0.0336 |  |  |  |  |  | 0.01 | 0.33 |
| Herb layer specialist richness |  | -0.142 |  |  |  |  | 0.06 | 0.42 |
| Herb layer generalist richness | -0.419*** |  |  | -0.111 | 0.041 |  | 0.10 | 0.74 |
| Herb layer proportion of specialist | 0.501*** | -0.206 |  |  |  |  | 0.02 | 0.05 |
| Herb layer CWM temperature index |  | -0.822*** |  |  |  |  | 0.70 | 0.81 |
| Herb layer CWM seed mass |  |  |  | 0.408 | 0.281 |  | 0.03 | 0.28 |
| Herb layer CWM height | -0.275 | -0.398 |  |  |  |  | 0.18 | 0.46 |
| Herb layer CWM sla |  | 0.482*** | 0.172 | -0.020 | -0.567* | | 0.28 | 0.54 |

**Table B3.** Summary of the results of generalized linear mixed models testing the influence of edaphic: (C:N, litter quality, % of sand and soil pH); climatic (microclimate soil temperature) and landscape (% forest cover= proportion of forest in a radius of 500 m and PAI=plant area index) conditions on the abundance and diversity of the herb layer. Variables not shown in the table were not included in any final model (MAP). Values are parameter estimates after model selection, positive or negative values denote the direction of the effect. The significance of p values is given with *** for p < 0.001; ** for p < 0.01; * for p < 0.05. Model fit was assessed based on marginal R^2^, the proportion of variance explained by fixed effects and conditional R^2^, the proportion of variance explained by both random and fixed effects (Nakagawa and Schielzeth, 2013). For variables explanation see Table A10.

|  | **PAI** | **C:N** | **Litter**  **quality** | **%**  **sand** | **Soil pH** | **Soil**  **temperature** | **% forest**  **cover** | **R2 marginal** | **R2 conditional** |
| --- | --- | --- | --- | --- | --- | --- | --- | --- | --- |
| Herb layer species richness | -0.174*** |  |  |  | 0.139** | |  | 0.19 | 0.73 |
| Herb layer.shannon |  |  |  |  | 0.251** |  |  | 0.12 | 0.55 |
| Herb layer evenness | 0.035 |  |  |  | 0.062** | |  | 0.10 | 0.48 |
| Herb layer specialist richness |  | -0.033 |  |  | 0.209** | |  | 0.18 | 0.39 |
| Herb layer generalist richness | -0.213*** |  |  |  |  | 0.198 |  | 0.16 | 0.76 |
| Herb layer proportion of specialist | 0.213** |  |  |  |  |  |  | 0.01 | 0.05 |
| Herb layer CWM temperature index |  |  |  | 0.163* |  |  | -0.11773 | 0.04 | 0.81 |
| Herb layer CWM seed mass |  | 0.068 |  |  |  |  |  | 0.03 | 0.36 |
| Herb layer CWM height |  | 0.212** | 0.45885* |  |  | 0.764 |  | 0.19 | 0.56 |
| Herb layer CWM sla |  | -0.540* |  | -1.187 | 1.563* | -3.559*** |  | 0.37 | 0.49 |


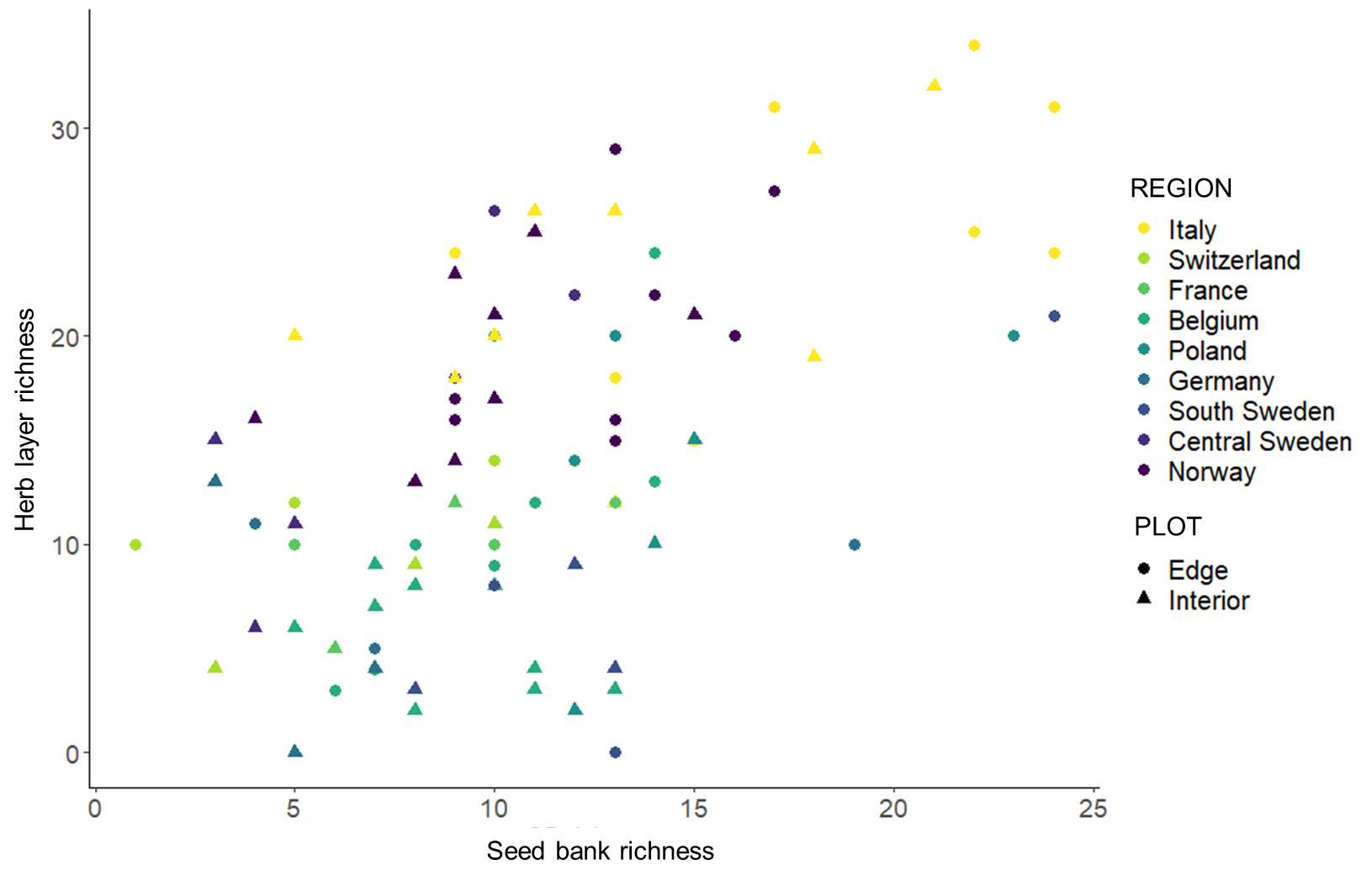


**Figure B2.** Correlation between soil seed bank richness and herb layer richness in 90 plots distributed in temperate deciduous forests along an edge vs interior, latitudinal, elevational and forest type gradients (dense, intermediate and open forests). In three regions, Norway, Belgium and Italy, soil samples were collected along an elevational gradient (low, intermediate and high elevation forests) while for the six other regions, the sampling was restricted to lowland forests. Shading corresponds to 95% confidence intervals.
